# Unraveling the mechanisms of triplet state formation in a heavy-atom free photosensitizer†

**DOI:** 10.1039/d4sc01369g

**Published:** 2024-04-02

**Authors:** Thomas P. Fay, David T. Limmer

**Affiliations:** a Department of Chemistry, University of California Berkeley CA 94720 USA tom.patrick.fay@gmail.com dlimmer@berkeley.edu; b Kavli Energy Nanoscience Institute Berkeley CA 94720 USA; c Chemical Science Division Lawrence Berkeley National Laboratory Berkeley CA 94720 USA; d Material Science Division Lawrence Berkeley National Laboratory Berkeley CA 94720 USA

## Abstract

Triplet excited state generation plays a pivotal role in photosensitizers, however the reliance on transition metals and heavy atoms can limit the utility of these systems. In this study, we demonstrate that an interplay of competing quantum effects controls the high triplet quantum yield in a prototypical boron dipyrromethene-anthracene (BD-An) donor–acceptor dyad photosensitizer, which is only captured by an accurate treatment of both inner and outer sphere reorganization energies. Our *ab initio*-derived model provides excellent agreement with experimentally measured spectra, triplet yields and excited state kinetic data, including the triplet lifetime. We find that rapid triplet state formation occurs primarily *via* high-energy triplet states through both spin–orbit coupled charge transfer and El-Sayed's rule breaking intersystem crossing, rather than direct spin–orbit coupled charge transfer to the lowest lying triplet state. Our calculations also reveal that competing effects of nuclear tunneling, electronic state recrossing, and electronic polarizability dictate the rate of non-productive ground state recombination. This study sheds light on the quantum effects driving efficient triplet formation in the BD-An system, and offers a promising simulation methodology for diverse photochemical systems.

## Abstract 2

Triplet excited states play a pivotal role in photosensitizers. Detailed simulations reveal how these states are formed efficiently in heavy-atom free systems.

## Graphical abstract

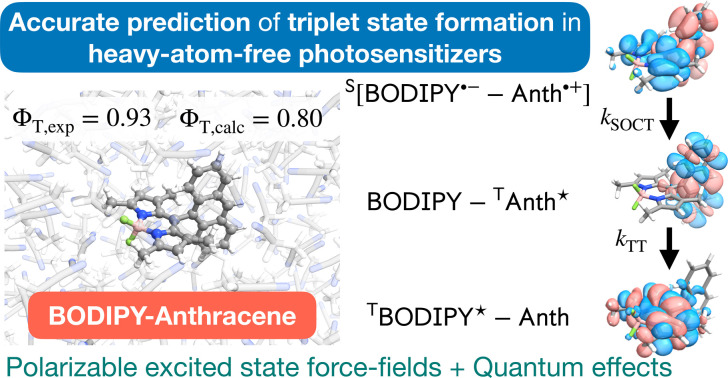

## I. Introduction

Photosensitizers harvest photons and transfer energy to other molecules, enabling new chemistry and photophysics, for applications ranging from photocatalysis,^1–4^ bioimaging,^5–7^ and photon upconversion.^8–11^ For photosensitizers to function efficiently, the electronic excitation needs to be generated in high yield and persist for a long time. One strategy to achieve this is to engineer the sensitizer to rapidly convert short-lived singlet excited states that are generated through photoexcitation into triplet excited states through intersystem crossing. Relaxation of triplet excited states to the singlet ground state is spin-forbidden, allowing the excitation to persist for orders of magnitude longer than in singlet excited states. In many photosensitizers, efficient intersystem crossing is facilitated by the presence of heavy atoms, such as transition metals, which enhance the spin–orbit coupling between singlet and triplet excited states. Recently, a large class of heavy-atom free triplet photosensitizers have been developed, capable of producing long-lived triplet excited states in high yields without the presence of heavy atoms.^12–16^ Understanding how triplet formation happens in these systems is essential for the design of other photocatalysts and photosensitizers. Using explicit molecular simulations of *ab initio* derived models, we reveal the mechanism by which triplet state formation occurs in a molecule made of only light elements.

**Fig. 1:**
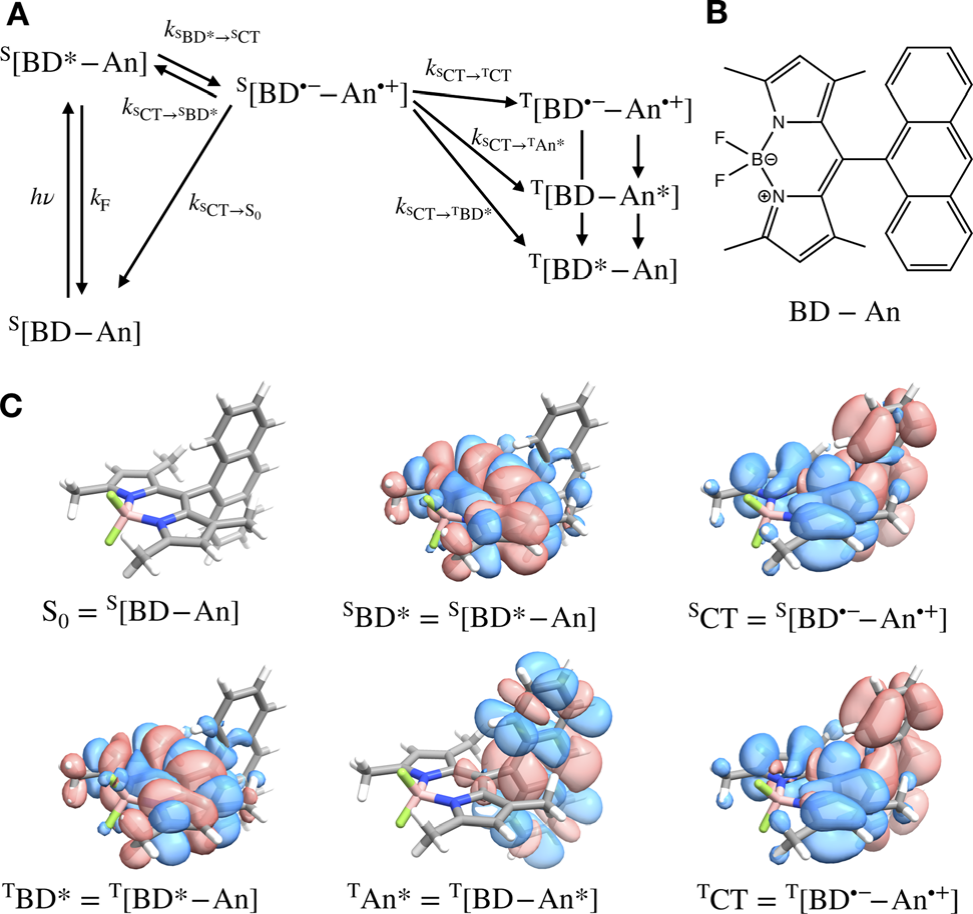
(A) Scheme showing the excited state interconversion processes we consider in this work. (B) The chemical structure of BD-An. (C) Difference densities for each of the excited states calculated at with TDDFT using the SOS-ωB2GP-PLYP functional and def2-TZVP(-f) basis set.

In this work we focus on a prototypical heavy-atom-free photocatalyst, the boron-dipyrromethene-anthracene (BD-An) dyad (chemical structure in Fig. 1B).^5,17–19^ BD-An has recently found applications in synthetic chemistry^20–22^ and its derivatives have been investigated for phototheraputic applications.^23^ The competing photophysical processes and the electronic excited states involved are summarized in Fig. 1. BD-anuses excited state charge transfer from an anthracenyl (An) group to the photoexcited ^S^BD* forming an ^S^CT state, to enable rapid triplet ^T^BD* formation with a high experimental yield, *Φ*_T_ = 0.93–0.96.^18,24^ Naively one might expect excited state charge transfer to reduce the triplet quantum yield, since the charge transfer state provides a charge recombination pathway for relaxation to the singlet ground state. However experiments indicate that charge recombination is suppressed by the large charge recombination free energy change, pushing this reverse electron transfer deep into the Marcus inverted regime, where increasing the free energy change increases the activation energy.^18^ This effect is captured qualitatively by Marcus' theory for the reaction rate constant^25,26^1
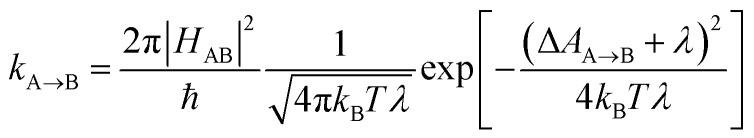
where *H*_AB_ is the coupling between electronic states A and B, Δ*A*_A⃑B_ is the free energy change of the reaction and *λ* is the reorganization energy, which encodes how solvent fluctuations and intramolecular vibrations control electronic state transitions, ℏ is Planck's constant and *k*_B_*T* is Boltzmann's constant times the temperature. Spin conserving charge recombination to the ground state is in the Marcus inverted regime, −Δ*A*_A→B_ ≫ *λ*, which requires a significant activation energy to proceed, whilst for the spin–orbit coupled charge transfer to the triplet excited state −Δ*A*_A→B_ ≈ *λ*, the reaction is approximately activation-less and thus this spin-forbidden process is competitive, despite *H*_AB_ being much smaller for the spin-forbidden charge recombination. However, Marcus theory is not accurate in the inverted regime due to significant nuclear quantum effects, and alternate triplet formation pathways *via* high-energy triplet states could contribute, as has been observed in TREPR studies wherein ^T^CT and ^T^An* intermediates were detected at low temperatures.^24^

We aim to investigate the efficiency of BD-An triplet state generation in solution, going beyond the Marcus picture through first principles computational and theoretical methods, in order to explain how spin-crossover competes with charge recombination and fluorescence in solution. To this end, we interrogate each of the photophysical processes outlined in Fig. 1A by combining electronic structure calculations, molecular dynamics simulations and non-adiabatic rate theories.^26,27^ Our aim is to develop models that quantitatively predict experimental observables and give physical insight into mechanisms of triplet formation. We find that effects not captured by Marcus theory, including nuclear tunneling and zero-point energy, have a large effect on the non-adiabatic reaction rate constants, and must be accounted for in our description of these systems.^27–30^ Furthermore, Marcus theory relies on weak coupling between charge transfer states that does not hold for some of the important processes in BD-An, which we investigate with numerically exact open-system quantum dynamics calculations.^31–34^

The importance of solvent effects poses a particular challenge in developing a first principle understanding of triplet state formation, because this necessitates the use of explicit solvent models and molecular dynamics.^27^ However common general force fields for organic molecules are only applicable to describe the ground electronic state of these systems. Previous studies have primarily used gas phase electronic structure calculations to rationalize observed behavior,^18,19^ but these have not attempted to quantitatively predict rate constants from first principles. To address these challenges, we have developed a protocol for excited state force field parameterization, enabling us to accurately describe solvent fluctuations that control charge transfer processes in ground and excited states. With these tools, we show that the photophysics of BD-An can be quantitatively predicted and mechanisms of triplet formation can be understood in detail. We start by providing a brief description of the computational methods used in this study. We then show our results for predicted spectra, free energy changes and rate constants, followed by a discussion of how these can be used to understand efficient triplet formation in BD-An.

## II. State energies and spectra

**Fig. 2:**
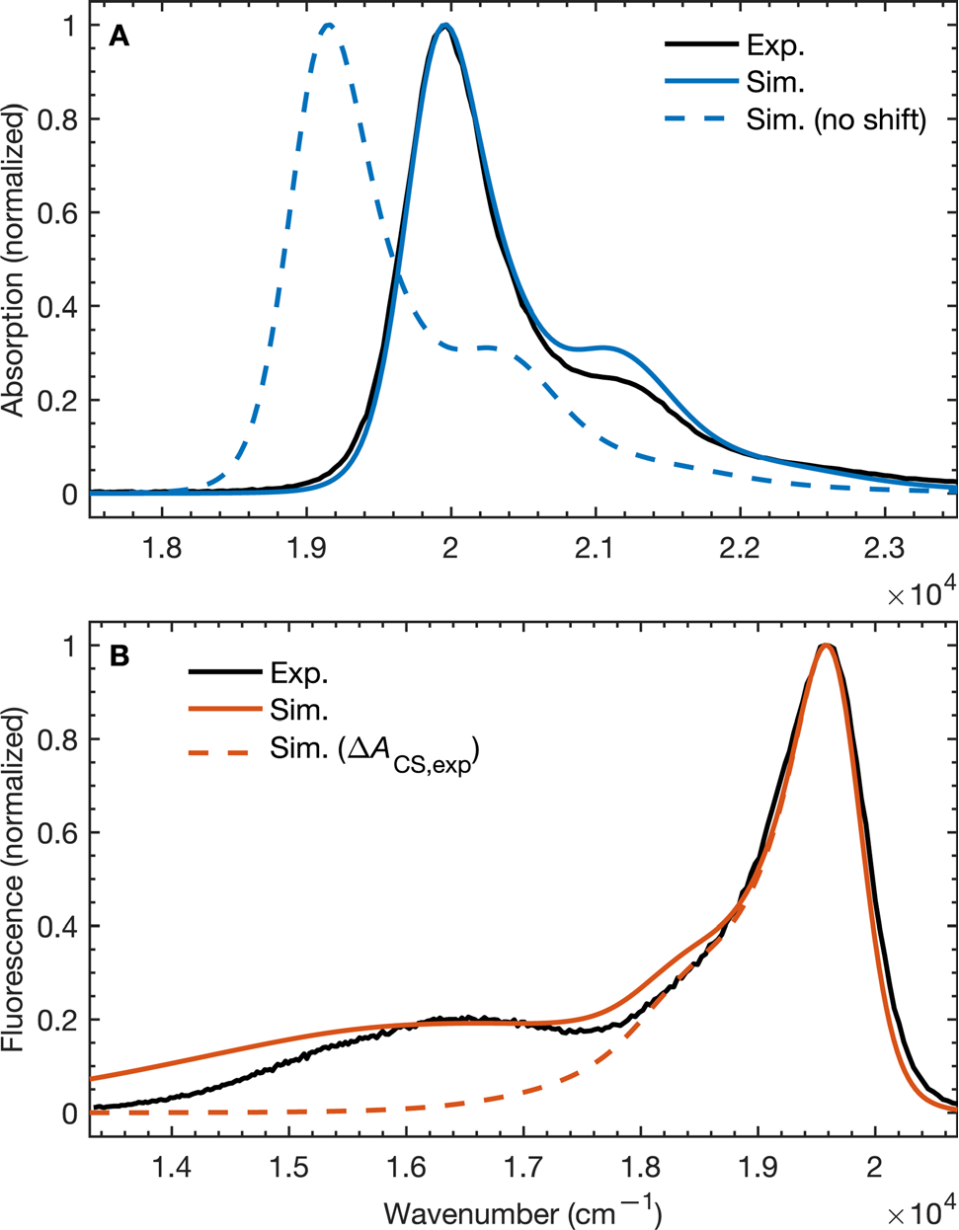
(A) Absorption and (B) emission spectra of BD-An comparing calculated and experimental spectra with and without shifts in the excited state energies. The simulated line-shapes are obtained from the spin-boson mapping described in the main-text with bespoke force-fields for the excited states. The energy differences between excited states were obtained from DLPNO-STEOM-CCSD/def2-TZVP(-f) calculations combined with solvation energies from molecular dynamics. Experimental spectra obtained from ref. 18.

To validate our molecular model, we have computed the BD-An absorption and fluorescence spectra (shown in Fig. 2). We calculated gas phase energies of the excited states using high-level wave-function based the DLPNO-STEOM-CCSD/def2-TZVP(-f) method^35,36^ (or DLPNO-CCSD(T)/def2-TZVP(-f) for the ^T^BD* and ^T^AN* states^37^), with geometries for each of the excited states obtained from TDA-TDDFT^38,39^ with the ωB97X-D3/def2-SVP functional^40^ and basis set.^41^ All calculations were performed with Orca 5.0.3.^42–44^ We found that wave-function based methods, which account for orbital relaxation in the excited state are required in order to obtain an accurate S_0_–^S^CT gap.

In the absence of solvation effects, the ^S^BD* state is lower in energy than the ^S^CT state by about 0.5 eV (see ESI† for list of energies), which is inconsistent with the fluorescence spectrum, which shows a clear peak from the CT state at lower energies than the ^S^BD* peak. Thus in order to predict solvation effects and spectral line-shapes, we constructed bespoke force-fields for the ground and excited states of BD-An, which enabled us to perform molecular dynamics simulations to efficiently compute spectra with the spin-boson mapping.^45^ Geometries and Hessians from TDA-TDDFT to were used to parameterize intramolecular force-fields^46,47^ based on the OPLS-AA force-field.^48,49^ Electronic polarizability was accounted for using the Drude oscillator model.^50^ We used the same procedure to parameterize both polarizable^50^ and non-polarizable force-fields for the acetonitrile (ACN) solvent, with further non-bonded parameter refinement targeting the dielectric properties of the solvent. The BD-An molecule was solvated in a box of 512 ACN molecules, and energy gap correlation functions were calculated from *NVE* trajectories, initial after *NPT* and *NVT* equilibration (full details are given in the ESI†).

From the molecular dynamics (MD) trajectories, the spin-boson mapping was constructed, from which spectra were then calculated.^45,51,52^ In this approach the full anharmonic potential energy surfaces *V*_J_ are mapped onto effective harmonic potential energy surfaces. Observables of this harmonic model are fully determined by the spectral density 
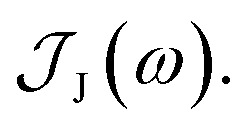
 We fit the spectral distribution 
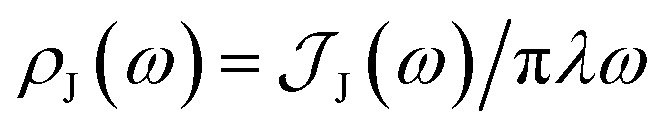
 from the energy gap correlation function obtained from molecular dynamics,^29^2
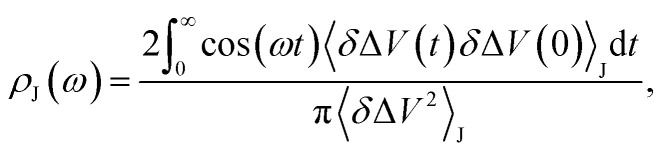
where Δ*V* = *V*_B_ − *V*_A_, *δ*Δ*V* = Δ*V* − 〈Δ*V*〉_J_ and 〈⋯〉_J_ denotes the classical phase space average over the equilibrium distribution for state J with dynamics calculated on the same surface. For the absorption spectrum we use dynamics on J = ^S^BD* and for the fluorescence spectra we use J = S_0_ to compute the mapping, and the reorganization energy *λ* is fit from free energy calculations using the same force fields (see below for details). From this mapping the spectra can be calculated from the Fourier transform of correlation function *c*_AB_(*t*), which is given by3
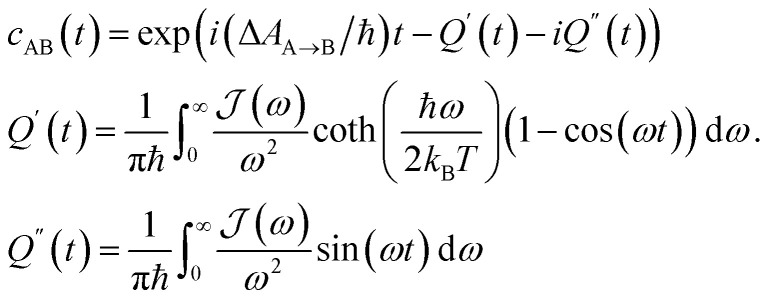


The absorption, *A*_J_(*ω*), and fluorescence, *F*_J_(*ω*), spectra (with unit area) are then given by4
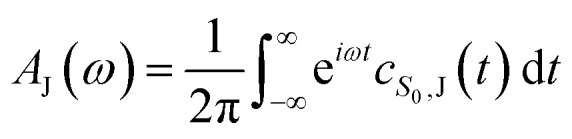
5
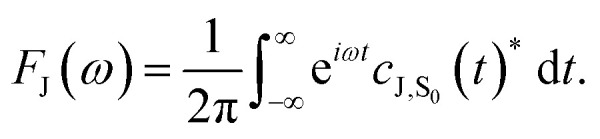


Further details of force-field development and the spin-boson mapping are provided in the ESI.†

The unshifted spectra calculated from the spin-boson mapping using DLPNO-STEOM-CCSD/def2-TZVP(-f) gas phase energy gaps are shown in Fig. 2 as dashed lines. Our calculated spectra show good overall agreement in the spectral line shapes, without any additional fitting, capturing the narrow ^S^BD* peak in the absorption and fluorescence spectra, including a small vibronic side band at about 1500 cm^−1^ from the main peak, as well as the broad ^S^CT fluorescence band. The agreement in the vibronic structure in the ^S^BD* peaks suggests the fitted force fields capture the reorganization energies between excited states relatively well. However we see that the unshifted absorption spectrum calculations underestimates the ^S^BD* energy, which we attribute to the fact that the triple zeta def2-TZVP(-f) basis set is likely still not sufficient for this system. As a result, we shifted all excited states by 805 cm^−1^ in order to fit the experimental absorption spectrum. This simple shift is justified by the fact that all excited states shift by ∼0.15 eV on increasing the basis set size from def2-SVP to def2-TZVP(-f), but differences between excited state energies change by much less (see ESI† for details). Furthermore it has been found the EOM-CCSD has typical errors of around 0.3 eV ≈ 2400 cm^−1^ for charge transfer states, so introducing a shift of 805 cm^−1^ seems justifiable. This shift is also used later in the free energy and rate calculations.

Using the shift from the absorption spectrum, the fluorescence spectrum (Fig. 2B) was calculated as a weighted sum of the ^S^CT and ^S^BD* emission spectra, with weights given by the transition dipole moments from DLPNO-STEOM-CCSD, *μ*_^S^BD*,S_0__^2^ = 7.59 a.u. and *μ*_^S^CT,S_0__^2^ = 0.54 a.u., and equilibrium populations of the two states given by the free energy change of charge separation Δ*A*_CS_, *i.e.*6
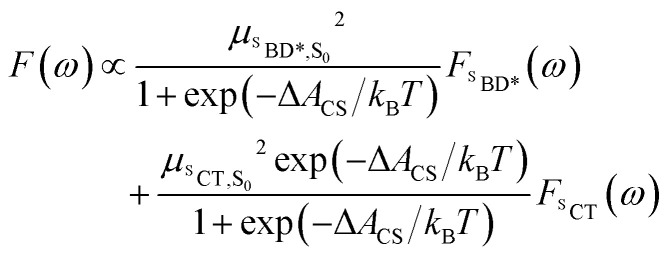


The assumption of equilibrium between the ^S^BD* and ^S^CT states is justified by the fact the time-scale of equilibration of these states is ∼10^3^ times shorter than the lifetime of these states (as we will discuss shortly). We have also computed the fluorescence spectrum assuming the populations of the ^S^BD* and ^S^CT states are given by the experimental estimate, Δ*A*_CS,exp_, based on the approximate Weller equation, which is about 0.2 eV larger than our estimate.^18^ Because Δ*A*_CS,exp_ > 0, the ^S^CT state is significantly less populated relative to the ^S^BD* state and the ^S^CT fluorescence peak is almost completely suppressed, which does not agree with the experimental spectrum. This suggests that the Weller equation cannot be used reliably when free energy changes are close to zero. As an interesting aside, the strongest ^S^CT–S_*n*_ coupling (see Table 1) is to the S_0_ state, by over a factor of 10, which indicates that the intensity borrowing effect responsible for the ^S^CT emission arises primarily from mixing between ^S^CT and S_0_ states, rather than ^S^CT and ^S^BD* states, as has previous been assumed.^24^

**Table 1:** Uncertainties in the simulated free energy changes and reorganization energies (2*σ*) are all <0.005 eV ≈ 0.2*k*_B_*T*

A	B	Calc. Δ*A*_A→B_ (eV)	Exp. Δ*A*_A→B_^b^ (eV)	*λ* c (eV)	|*H*_AB_|^d^ (cm^−1^)	*k*_A→B_ e (s^−1^)
^S^BD*	^S^CT	−0.057 ± 0.005	+0.13	0.550 ± 0.002	99	(1.46 ± 0.04) × 10^11^
^S^BD*	S_0_	−2.4542 ± 0.0004	−2.460	(8.77 ± 0.08) × 10^−2^ f	—	(1.0747 ± 0.0006) × 10^8^ g
^S^CT	S_0_	−2.397 ± 0.003	−2.59	0.483 ± 0.003	1904	(3.4 ± 0.5) × 10^7^ h/(3.6 ± 0.6) × 10^7^^i^
^S^CT	^T^BD*	−0.826 ± 0.005	−0.97	0.584 ± 0.001	0.79	(7.9 ± 0.2) × 10^7^
^S^CT	^T^AN*	−0.524 ± 0.004	—	−0.477 ± 0.002	0.63	(9.7 ± 0.1) × 10^7^
^S^CT	^T^CT	−0.112 ± 0.001	—	−0.119 ± 0.002	0.21	(2.86 ± 0.02) × 10^7^
^T^AN*	^T^BD*	−0.302 ± 0.003	—	0.565 ± 0.002	2.57	(1.09 ± 0.01) × 10^9^
^T^BD*	S_0_	−1.638 ± 0.001^a^	−1.62^j^	0.512 ± 0.002^k^	0.19	(1.045 ± 0.006) × 10^4^ k

a Free energy changes calculated with non-polarizable ACN, from thermodynamic integration/MBAR.

b Estimated free energy changes from ref. 18 calculated with the Rehm–Weller equation Δ*A* ≈ Δ*G* = *e*(*E*_D_ − *E*_A_) − ΔE* − *e*^2^/(4π*ε*_0_*ε*_r_*r*_DA_).

c Reorganization energies from equating the *p*^Gaussian^_J_(*ε* = 0) with *p*_J_(*ε* = 0) (see ESI for details).

d Couplings averaged over gas-phase equilibrium geometries of A and B, |*H*_AB_|^2^ = (|*H*_AB,A_|^2^ + |*H*_AB,B_|^2^)/2. Details of calculations given in ESI. Ref. 18, estimated from spectroscopic measurements.

e Rate constants from spin boson mapping.

f Linear response value: *λ* = (〈Δ*V*〉_B_ − 〈Δ*V*〉_A_)/2.

g Radiative rate constant (eqn (9)).

h With recrossing correction and.

i Without recrossing correction.

j Estimated from spectroscopic measurements.^18^

k Using reorganization energy from non-polarizable umbrella sampling calculations (see ESI).

## III. Charge separation and recombination

### A. Thermodynamics

Charge separation, the ^S^BD* → ^S^CT process, and charge recombination, the ^S^CT → S_0_ process, both play an important role in efficient triplet formation. Efficient charge separation is required to suppress fluorescence from the ^S^BD* state, but slow charge recombination is needed to enable intersystem crossing to occur to generate triplet states. From our excited state force-fields, we have calculated free energy changes associated with these processes from molecular dynamics and the multi-state Bennett acceptance ratio (MBAR).^53,54^ As discussed above, the calculated free energy change for charge separation is −0.057 eV, thus population of the ^S^BD* state is reduced and fluorescence is suppressed.

We have also calculated the rates of these processes from the same MD simulations, by calculating the probability of two states being at resonance. This probability controls the classical Fermi's Golden rule (FGR) rate for the transition between A and B.^55^ The free energy along the energy gap coordinate, Δ*V* = *V*_B_ − *V*_A_, is related to the energy gap distribution *p*_J_(*ε*) = 〈*δ*(Δ*V* − *ε*)〉_J_ by^27^

**Fig. 3:**
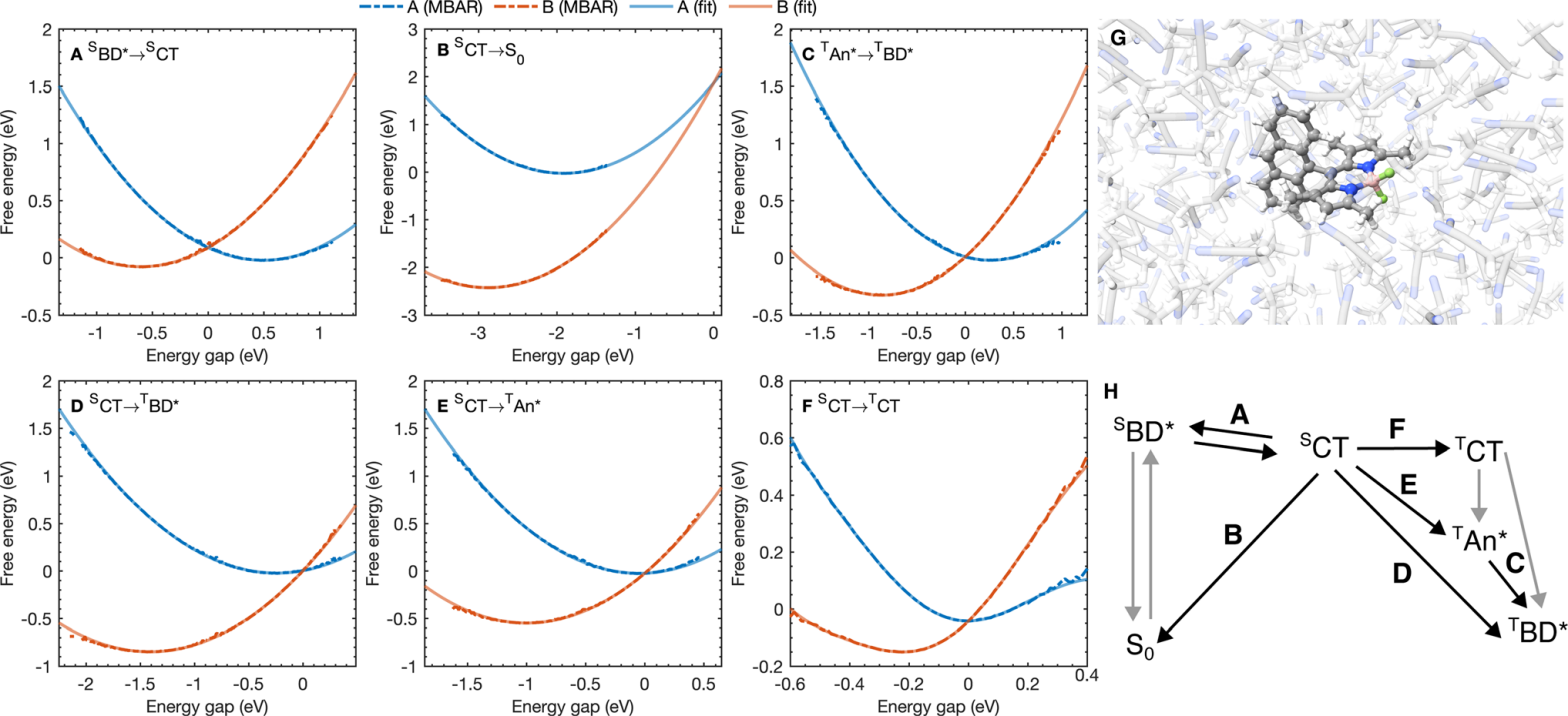
(A–F) Free energy curves for the six A → B processes considered with the reaction A → B labeled on each figure. Points correspond to free energy curves calculated with MBAR and lines correspond to polynomials fitted to the MBAR cumulative distribution functions (see ESI† for details). (G) A snapshot for molecular dynamics simulations on the S_0_ potential energy surface. (H) A scheme highlighting the processes in (A–F).

7 *A*_J_(*ε*) = −*k*_B_*T*ln(*p*_J_(*ε*)) + (*A*_B_ − *A*_J_)

for J = A or B. In Fig. 3 we show the free energy profiles calculated from MD simulations on each of excited state surfaces with the polarizable ACN model using MBAR. The crossing point of the two curves gives the free energy barrier for the transition, which dictates the classical FGR rate, *k*^class^_A⃑B_ = (2π/ℏ)|*H*_AB_|^2^e^−*A*_A_(*ε*=0)/*k*_B_*T*^.^27^ If the free energy curve is perfectly quadratic, then this reduces exactly to Marcus theory [eqn (1)].^25,26,29^ For the charge recombination the crossing point occurs outside of the sampled region, so we extrapolated to the crossing point using a quadratic polynomial ansatz for the free energy, fitted to the cumulative distribution function. This procedure was found to result in very little loss in accuracy when compared to umbrella sampling/weighted histogram analysis^56^ calculations performed using the non-polarizable ACN model (see ESI† for details).

The free energy curves for charge separation and charge recombination are shown in Fig. 3A and B, where we see charge separation lies in the Marcus normal regime, whereas charge recombination is deep in the Marcus inverted regime, with a much larger free energy barrier. Using diabatic state couplings calculated from the generalized Mulliken–Hush method^57^ with DLPNO-STEOM-CCSD calculations, we can directly calculate the classical FGR rates for these processes (couplings |*H*_AB_| are shown in Table 1). The classical FGR charge separation rate is 4.8 × 10^10^ s^−1^, about a factor of 10 smaller than the experimentally observed rate of 5.4 × 10^11^ s^−1^, however the charge recombination rate is predicted to be 1.1 × 10^−17^ s^−1^, which is more than 10^24^ times too small compared to the experimental estimate of 2.3 × 10^7^ s^−1^.^18^ This enormous discrepancy can be attributed to nuclear quantum effects, in particular the important role of nuclear tunneling in the inverted regime.

### B. Quantum effects on rates

In order to include nuclear quantum effects in the rate calculations, we employed the same spin-boson mapping approach as was used to compute the spectra. The full FGR rate constant is given by8
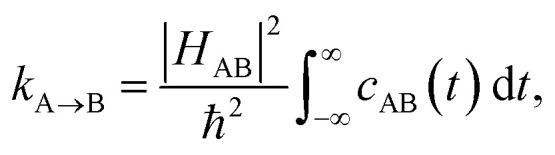
which can be evaluated directly using eqn (3). The reorganization energy *λ* is fitted by requiring that the classical limit of the spin-boson mapping reproduces the exact classical limit rate constant, obtained from the classical free energy barrier calculated from MBAR.^54^ This approach to calculating the rate can be regarded as a generalization of the commonly used Marcus–Levich–Jortner theory, accounting for the full frequency dependence of the reorganization energy, which is encapsulated in *ρ*_J_(*ω*). The final rate constant is obtained as a simple average over the rate constants calculated with spectral distributions *ρ*_A_(*ω*) and *ρ*_B_(*ω*).

**Fig. 4:**
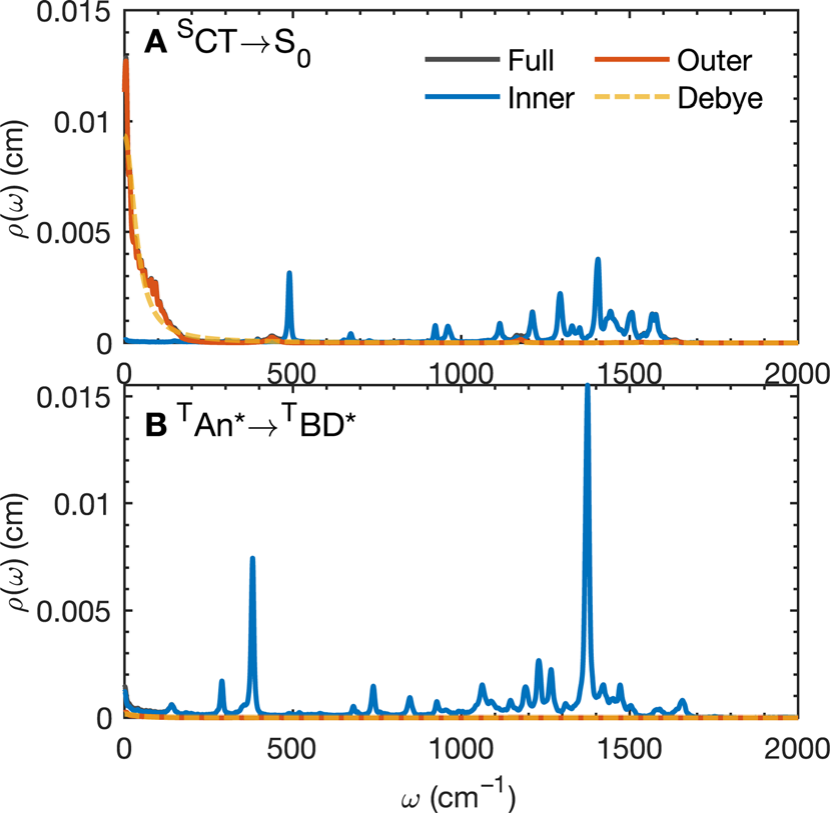
Spectral distribution *ρ*(*ω*) for (A) the ^S^CT → S_0_ process computed from dynamics on the ^S^CT potential energy surface and (B) the ^T^AN* → ^T^BD* process computed from dynamics on the ^T^AN* potential energy surface. The decomposition into inner and outer sphere contributions and the Debye approximation for the outer-sphere component is also shown, *ρ*_D_(*ω*) = (2/π)/(1 + *τ*_D_^2^*ω*^2^), where *τ*_D_ = (*ε*_*∞*_/*ε*_r_)*τ*_rel_, and *τ*_rel_ is the solvent dipole–dipole autocorrelation relaxation time, and *ε*_∞_/*ε*_r_ are the optical and static dielectric constants of the ACN model.

The calculated spectral distributions *ρ*_J_(*ω*) can be decomposed into inner sphere, outer sphere and cross-correlated contributions, by decomposing the energy gap into molecular and the remaining environment contributions Δ*V* = Δ*V*_mol_ + Δ*V*_env_. We find that the cross-correlated contribution is generally negligible for all processes in BD-An, so the reorganization energy is well-described by a simple sum of inner and outer sphere contributions. The inner and outer sphere spectral distributions are calculated with the non-polarizable ACN/solute model, with the outer sphere contribution scaled down to match the polarizable model outer sphere contributions. As can be seen in Fig. 4A, the low frequency proportion of the spectral distribution for the ^S^CT → S_0_ transition is dominated by the outer sphere contribution arising from solvent molecule fluctuations, making up ∼50% of the reorganization energy, which is well approximated by the Debye model.^32^ In contrast, the high frequency region of the spectral density is dominated by the inner sphere contribution from changes in equilibrium bond lengths in the BD-An molecule on charge transfer. The inner sphere spectral distribution has contributions over a range of frequencies from around 500 to 1600 cm^−1^, all of which contribute to tunneling enhancement of the ^S^CT → S_0_ rate, although the dominant mode at ∼1400 cm^−1^ likely corresponds to a C=C stretching motion within the aromatic rings. Qualitatively similar spectral distributions were found for the other charge transfer processes. For processes which do not involve charge transfer the spectral distribution is dominated by the inner sphere contribution, as can be seen for the ^T^AN* → ^T^BD* process in Fig. 4B.

When accounting for nuclear quantum effects, the ^S^BD* → ^S^CT rate goes up by a factor of ∼3 to 1.46 × 10^11^ s^−1^, and the ^S^CT → S_0_ rate goes up by over 10^24^ to 1.0 × 10^8^ s^−1^, and both calculated rates are now much closer to the experimentally measured values, agreeing much better with the experimental value. Application of Marcus–Levich–Jortner theory with the same inner and outer sphere reorganization energies and a characteristic inner-sphere frequency of 1500 cm^−1^ also predicts about a 10^24^-fold increase in the rate constant, compared to Marcus theory. This suggests that the large increase is robust to the details of the spectral density. Electronic polarizability is essential to account for in calculating the charge recombination rate. When a non-polarizable model is used instead, the free energy change of the reaction is effectively unchanged but the reorganization energy goes up by nearly 0.1 eV. This lowers the activation energy and accelerates the rate by around a factor of three.

**Fig. 5:**
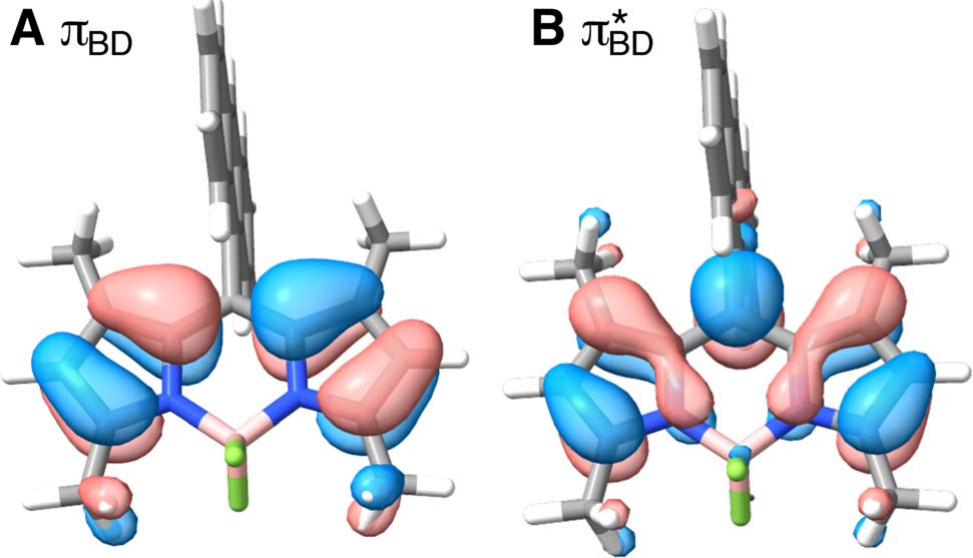
BD orbitals involved in charge separation and recombination (A) π_BD_ and (B) 
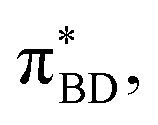
 calculated with ωB97X-D3/def2-TZVPP/CPCM(ACN) at the S_0_ equilibrium geometry.

**Fig. 6:**
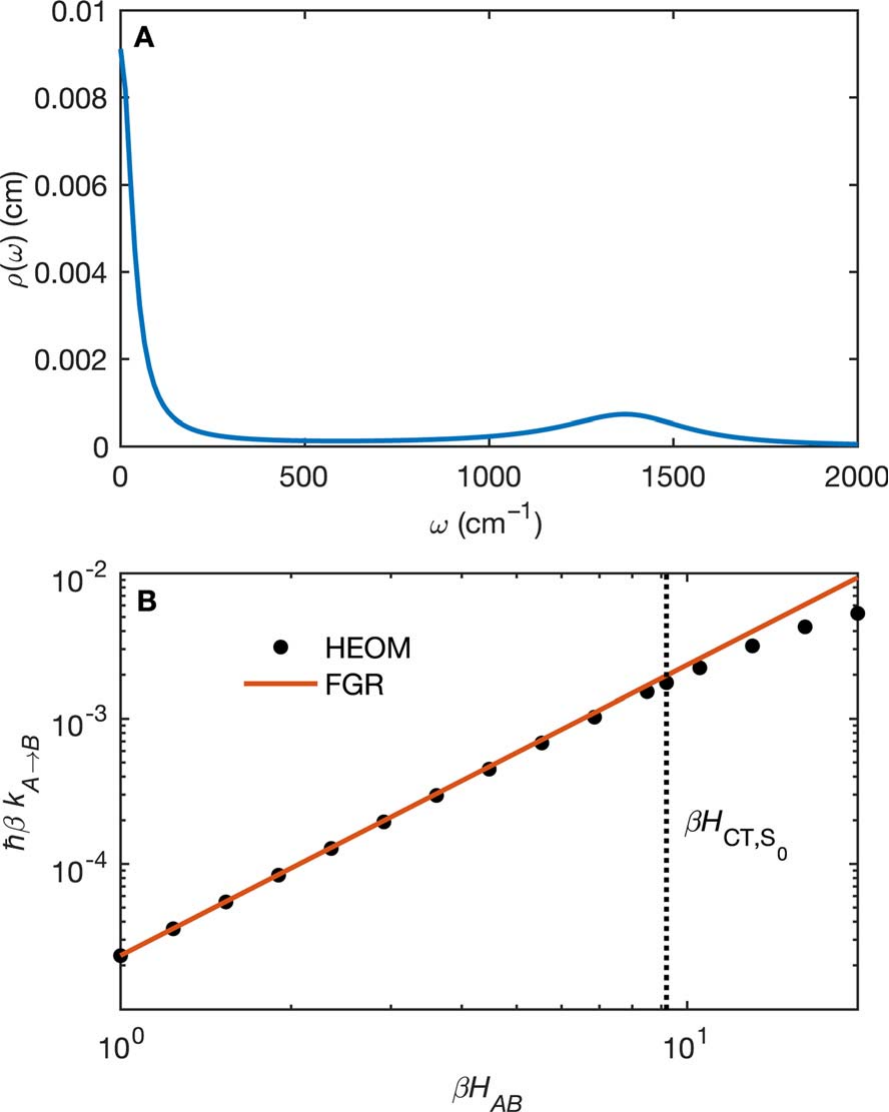
(A) The coarse-grained model spectral distribution for the ^S^CT → S_0_ transition, consisting of a low frequency Debye contribution *ρ*_D_(*ω*) = (1/2π)/(1 + (*ω*/*ω*_D_)^2^), with *βω*_D_ = 0.1831, and an under-damped Brownian oscillator contribution *ρ*_BO_(*ω*) = (1/2π)*γΩ*^2^/((*ω*^2^ − *Ω*^2^)^2^ + *γ*^2^*ω*^2^) with *βγ* = 4 and *βΩ* = 6.76. The reorganization energy for the Brownian oscillator portion is *βλ* = 8.6780 and for the Debye portion is *βλ* = 10.1459. (B) The rate constant from HEOM calculations for the coarse-grained spectral density as a function of *H*_AB_ together with the FGR predictions. The value of *H*_AB_ for the ^S^CT → S_0_ transition is also indicated. Calculations were performed using the heom-lab code^58^ using the HEOM truncation scheme from ref. 59.

Care should however be taken when using FGR to calculated the charge recombination rate. This is because the diabatic coupling for charge recombination process, *H*_AB_ = 1904 cm^−1^, is about 20 times larger than *k*_B_*T*, and thus higher-order diabatic coupling effects beyond FGR, may be important (although large nuclear quantum effects in the FGR rate have been observed to reduce the importance of higher order effects).^34^ The large difference in couplings arises from the BD π orbitals involved in the transitions. The ^S^BD* → ^S^CT coupling involves an interaction between π_An_ and π_BD_ (Fig. 5A) orbitals, whereas ^S^CT → S_0_ coupling involves the π_An_ and 
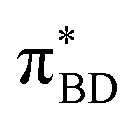
 (Fig. 5B) orbitals. As can be seen in Fig. 5 the π_BD_ has minimal density on the carbon atom bonded to the An, group, whereas the 
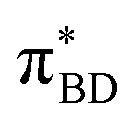
 orbital does. In order to investigate the potential role of higher-order diabatic coupling effects in the ^S^CT → S_0_ transition, we have performed Hierarchical Equations of Motion (HEOM) calculations a simple model for this transition. The spectral density for the transition is coarse-grained down to a low-frequency outer-sphere portion described with a Debye spectral density and the inner sphere portion is described with a single under-damped Brownian oscillator spectral density, with a characteristic frequency of 1400 cm^−1^. The coarse-grained spectral density is shown in Fig. 6A. For this simplified model the exact open quantum system dynamics can be obtained using the HEOM method, and from this the rate constant as a function of *H*_AB_ can be obtained. These rates are shown in Fig. 6B. We see that the rate constant is still fortuitously very well described by Fermi's Golden rule for this model, with only a factor of ∼0.9 reduction in the rate constant at the calculated value of *H*_AB_. We include this as a correction to the Fermi's Golden rule *k*_SCT⃑S0_ that we calculated with the full spectral density.

Radiative recombination from the ^S^CT state can also occur in BD-An, either through thermally activated delayed fluorescence *via* the ^S^BD* state, or directly. The radiative rates can be calculated from the fluorescence spectra obtained from the spin-boson mapping as^26,60^9
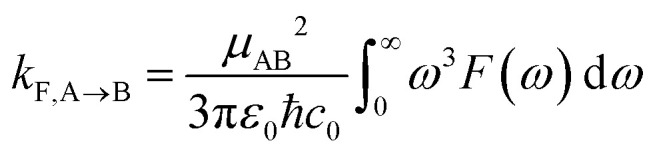
where *F*(*ω*) is the fluorescence line-shape computed from the spin-boson mapping. From this we find the fluorescence rate from the ^S^BD* state to be 1.1 × 10^8^ s^−1^ and the fluorescence rate from the ^S^CT state to be 7.7 × 10^8^ s^−1^. Assuming a pre-equilibrium between the ^S^BD* and ^S^CT states, as is justified by the large charge separation rate constant, we find that only 63% of the S_0_ re-formation occurs by direct non-radiative recombination, with 15% of recombination events happening by radiative ^S^CT recombination and 22% occurring *via*^S^BD* thermally activated delayed fluorescence.

## IV. Triplet state formation and lifetime

As with the charge separation and charge recombination processes, we have calculated the free energy changes and free energy profiles for the three triplet formation pathways: from the ^S^CT state to the ^T^CT, ^T^AN* and ^T^BD* states (Fig. 3D–F). Free energy calculations reveal that the three pathways are thermally accessible, with all three states lying lower in energy than the ^S^CT state. Furthermore all three pathways are approximately activation-less, which is at first surprising given that each process has a very different free energy change. The ^T^BD* pathways has a larger |Δ*A*_A→B_|, than the ^T^AN* pathway, but the ^T^AN* pathway has smaller inner and outer sphere reorganization energies, so this pathway is also approximately activation-less. The ^T^CT pathway has a very small reorganization energy which is dominated (∼90%) by the inner sphere contribution, at only 0.11 eV. This is because the ^S^CT and ^T^CT states have the same orbitals occupied, so the reorganization energy is dictated only by differences in the exchange energy which alters bonds lengths. However the net exchange energy difference between these states is small because the unpaired electrons have low spatial overlap, so overall the reorganization energy is low and this transition is approximately activation-less. Much like the spin-conserving charge separation and charge recombination, about 50% of the reorganization energies for the charge transfer processes is outer sphere, with the remaining 50% arising from inner sphere reorganization, although there is a significant range of reorganization energies for the charge transfer processes, from 0.48 eV to 0.58 eV. In contrast, the reorganization energies of processes which do not involve charge transfer are dominated by the inner sphere contribution, 89% for the ^S^CT → ^T^CT spin-crossover and 99% for the ^T^AN* → ^T^BD* triplet–triplet energy transfer, as illustrated in Fig. 4B. The triplet–triplet energy transfer still has a reorganization energy comparable to the charge transfer processes, at 0.57 eV, due to a large change in bond order in both the BD and An units in this process. Further analysis of the inner/outer sphere reorganization energies are given in the ESI† together with all calculated spectral densities.

We have also calculated the SOC couplings between the different ^S^CT and triplet states using TDDFT (ωB97X-D3/def2-TZVPP/CPCM(ACN)) and the spin–orbit mean-field (SOMF) treatment of spin–orbit coupling.^61,62^ The two spin–orbit coupled charge transfer (SOCT) pathways have the largest SOC couplings, at 0.79 cm^−1^ and 0.63 cm^−1^ for the ^T^BD* and ^T^AN* whilst the formally El-Sayed's rule forbidden pathway has a smaller coupling at 0.21 cm^−1^. Using these couplings and the spin-boson mapping, we find that two El-Sayed's rule allowed transitions, *via*^T^AN* and ^T^BD*, occur at very similar rates, with ^S^CT → ^T^BD* occurring only about 20% faster than the ^S^CT → ^T^AN* formation. The triplet–triplet ^T^AN* → ^T^BD* energy transfer is also activation-less (Fig. 3C), and has a coupling from fragment energy/charge density (FED/FCD) calculations^47,63,64^ of 2.57 cm^−1^, and so occurs about 10 times faster than the triplet formation rate, accelerated by a factor of 1.6 by nuclear quantum effects, so the steady state population of ^T^AN* would be difficult to observe spectroscopically at room temperature. The El-Sayed's rule forbidden transition to the ^T^CT state also contributes to triplet formation, although it occurs about 4.5 times slower than ^T^BD* formation. The ^T^CT state very rapidly recombines to the ^T^AN* or ^T^BD* states, with these spin allowed transitions occurring at least ∼10^4^ times faster than the corresponding spin-forbidden transitions, so the ^T^CT state would be very difficult to observe directly at room temperature. Overall the ^T^CT ^T^AN*, and ^T^BD* pathways contribute 14%, 47%, and 39% respectively to the overall triplet formation. Surprisingly the most significant pathway is the ^T^AN* pathway and not the direct ^T^BD* pathway, which can be rationalized by the lower activation barrier for the ^T^AN* spin–orbit coupled charge recombination. The observation is consistent with TREPR experiments in which all three triplet states were observed, although at much lower temperatures (80 K) in a very different medium (a dichloromethane/isopropanol solid matrix). This work shows that multiple triplet formation pathways, including those forbidden by El-Sayed's rule, can contribute at room temperature in polar solvents. The presence of multiple triplet recombination pathways may also explain the large spread of effective spin–orbit coupled charge transfer rates observed in the family of BD-Aryl molecules studied in ref. 18.

Using all of the computed rates, we have estimated the observed charge separation and charge recombination rates, as well as the triplet yield. The effective charge separation rate corresponds to the observed equilibration rate between ^S^BD* and ^S^CT states *i.e. k*_CS,eff_ = *k*_^S^BD*→^S^CT_ + *k*_^S^CT→^S^BD*_. Likewise the effective charge recombination rate corresponds to the observed decay rate of the ^S^CT state, which under a pre-equilirbium approximation for the ^S^BD* ⇌ ^S^CT interconversion is given by

10 *k*_CR,eff_ = *p*_^S^CT_(*k*_CR_ + *k*_F,^S^CT_→_S_0__) + *p*_^S^BD*_k_F,^S^BD*→S_0__

where *p*_^S^CT_ = 1 − *p*_^S^BD*_ = *K*_CS_/(1 + *K*_CS_), with 
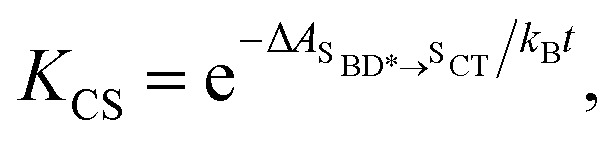
*k*_F,^S^BD*_ is the calculated fluorescence rate from the ^S^BD* state back to the S_0_ state and *k*_CR_ is the total recombination rate from the ^S^CT state, *i.e.*

11 *k*_CR_ = *k*_^S^CT→S0_ + *k*_^S^CT→^T^CT_ + *k*_^S^CT⃑^T^AN*_ + *k*_^S^CT^T^→BD*_.

The triplet quantum yield *Φ*_T_ is calculated as *Φ*_T_ = *p*_^S^CT_(*k*_^S^CT→^T^CT_ + *k*_^S^CT→^T^AN*_ + *k*_^S^CT→^T^BD*_)*τ*_CR_, with *τ*_CR_ = 1/*k*_CR,eff_, and the fluorescence yield *Φ*_F_ is *Φ*_F_ = *p*_^S^BD*_k_F,^S^BD*_τ_CR_. We also computed the fraction of non-radiative transitions which produce a triplet state, *ϕ*_CRT_ = *Φ*_T_/(1 − *Φ*_F_), as measured in ref. 18.

The calculated and experimental values of the rates and yields are summarized in Table 2. Overall we see excellent agreement between the calculated rates/yields and the experimental measurements from ref. 18 and 24, with less than a factor of 4 error in the charge separation rate and only a factor of ∼1.6 error in the charge recombination rate. Similar we only slightly underestimate the triplet yield, with our calculations yielding 0.80, compared to the experimental measurements between 0.93 and 0.96. If we only included the dominant ^S^CT → ^T^BD* triplet formation pathway, the triplet quantum yield would only be ∼0.6, and the error in the rate would be over a factor of 3. We also find that suppression of the charge recombination also plays a large role in efficient triplet formation, which is facilitated by polarizability and recrossing effects. Without including electronic polarizability, the charge recombination rate would be enhanced to ∼1.0 × 10^8^ s^−1^, which would reduce the triplet quantum yield to ∼0.63. This corroborates the conclusions drawn in ref. 18, although we find that multiple triplet pathways also enable the triplet formation to compete with charge recombination, which is suppressed by several effects. The net fluorescence quantum yield from ^S^BD* that we calculate, 0.045, is also in good agreement with the experimental values, between 0.01 and 0.018. These results suggest that the intersystem crossing rates are being slightly underestimated by our models, possibly due to errors in the reorganization energies or the spin–orbit couplings obtained from TD-DFT, which are all less than 1 cm^−1^.

**Table 2:** Calculated and experimental rates, quantum yields and triplet lifetime for the photophysics of BD-An

	*k*_CS,eff_ (s^−1^)	*k*_CR,eff_ (s^−1^)	*ϕ*_CRT_	*Φ*_T_	*Φ*_F_	*τ*_T_ (μs)
Calculated	(1.46 ± 0.04) × 10^11^	(2.31 ± 0.05) × 10^8^	0.86 ± 0.02	0.80 ± 0.02	0.045 ± 0.001	95.7 ± 0.6
Experiment [ref. 18]	5.4 × 10^11^	3.8 × 10^8^	0.94	0.93	0.01	—
Experiment [ref. 24]	—	3.3 × 10^8^	0.98	0.96	0.018	78

The triplet lifetime *τ*_T_ = 1/*k*_^T^BD*→S_0__ plays an important role in determining the utility of a triplet sensitizer or photocatalyst, with longer-lived triplet states allowing more time for diffusive encounters with other molecules enabling more efficient energy transfer. We have also calculated the triplet lifetime for BD-Anusing the methods described above, and we also find good agreement between our calculated value for *τ*_T_ and experimental measurements (Table 2), with an error of only ∼20%. From a simulation perspective, this requires an accurate calculation of the free-energy barrier, which requires enhanced sampling since the transition is very deep in the Marcus inverted regime, since it displays a highly non-quadratic free energy curve. This was achieved using the non-polarizable model with umbrella sampling^65^ on the energy gap coordinate Δ*V* sampled with the Fast-Forward Langevin algorithm.^66^ Use of the non-polarizable model is justified because over 99% of the reorganization energy is inner sphere for both ACN models, and solvent polarizability has less than a 1 meV effect on the free energy of the ^T^BD* state. As with the spin-conserving charge recombination, because the transition is deep in the inverted regime and the spectral distribution is dominated by high frequency inner sphere contributions, there is a very large nuclear quantum effect of over 10^7^ in the rate constant. One significant source of uncertainty in this is the validity of the spin-boson mapping, where rates calculated from the spectral distribution obtained from ^T^BD* and S_0_ dynamics vary by about 50%. This means that methods that more rigorously account for asymmetry and anharmonicity in the potential energy surfaces, while also accounting for nuclear quantum effects, may be needed to more accurately compute triplet lifetimes for this system and other related systems.^55,67,68^ However given the simplicity of the spin-boson mapping and its accuracy in this case, it is clearly still useful in prediction of non-adiabatic rates.

## V. Concluding remarks

Through this study, we have found that triplet formation in the photosensitizer BD-An hinges on a subtle balance of effects. Firstly charge separation occurs efficiently, which suppresses radiative decay from the ^S^BD* state. Secondly multiple triplet recombination pathways can operate, due to the range of reorganization energies and free energy changes associated with the rate-limiting intersystem crossing steps in each pathway, and in fact the high-lying triplet pathways make-up the major contribution to triplet formation, rather than direct SOCT to the ground triplet state. Thirdly, spin-conserving charge recombination to the S_0_ state is slowed down a high free energy barrier, with the transition being deep in the inverted regime, as well as diabatic recrossing effects, a significant portion of which arises due to electronic polarizability. The ^S^CT state energy plays an important role in triplet formation, since an increase in energy would increase fluorescence from ^S^BD*, but a decrease in its energy would reduce the barrier for spin-conserving charge recombination because this process is in the Marcus inverted regime. Capturing all of these effects depends on a complete description of the photophysics including accurate calculations of electronic state couplings, explicit solvent fluctuations, polarizability, to capture outer sphere reorganization energies, as well as an accurate description of molecular potential energy surfaces and inner sphere contributions to reorganization energies, as well as the nuclear quantum effects arising due to high frequency vibrations, which accelerate some processes by many orders of magnitude. Enhanced sampling techniques are also necessary to obtain accurate free energy barriers for important processes, namely the triplet decay.

The simulation techniques and bespoke force-field parametrization approach developed here paves the way for a quantitative modeling of other triplet photosensitizers and related systems,^69^ possibly even enabling straightforward computational screening for properties such as the triplet lifetime. Comparison between simulated and experimental optical spectra indicates that a major source of error is in gas phase energies of excited states. Even the popular wave-function-based DLPNO-STEOM-CCSD method appears to significantly underestimate transition energies, although the ground-state DLPNO-CCSD(T) method which can be used to calculate the T_1_–S_0_ gap seems robust. We also note that whilst the approximate spin-boson mapping seems fairly reliable for these systems, its application to deep inverted regime processes requires scrutiny. Thus BD-Ancould provide an interesting test-bed for recently developed approaches to calculating non-adiabatic transition rates applicable to high-dimensional anharmonic systems.^34,55,67,68,70–79^ The ^S^CT → S_0_ transition poses a particular challenge, since it is deep in the inverted regime, nuclear quantum effects are very large and strong diabatic coupling means there may be some effects missed by FGR, which we have estimated using open quantum dynamics simulations. Furthermore in this study we have neglected non-Condon effects^80^ and potential spin-vibronic effects,^81^ which could also play a role in determine the rates of conversion between excited states in this system. Future investigations into these potential effects could provide further insight into triplet formation in BD-An.

Overall, we believe the mechanistic insights gained from this study, which would be difficult to probe directly with experiment alone, could help light the path towards the development of novel and interesting photochemistry in related systems. The observation that high-energy triplet pathways dominate at room temperature opens the door to the intriguing possibility of engineering triplet anti-Kasha's rule systems,^82^ in which higher energy triplet states could be used to drive photochemistry. This could be particularly promising since triplet-triplet energy transfer is strongly distance dependent,^83^ so spatial separation of chromophore units could be used to extend the lifetime of high-lying triplet states. In summary, our comprehensive study highlights the intricate balance of factors influencing triplet formation, including the significance of charge separation efficiency, multiple recombination pathways, and nuclear quantum effects. Moving forward, this mechanistic understanding could steer the development of novel photochemical systems, with a wide range of potential applications.

## Abbreviations

ACN: Acetonitrile
An: Anthracene
BD: BODIPY, boron dipyrromethane
CPCM: Conductor-like polarizable continuum
CR: Charge recombination
CS: Charge separation
CT: Charge transfer
DLPNO-STEOM-CCSD: Domain local pair natural orbital similarity transformed equation of motion coupled cluster singles and doubles
DLPNO-CCSD(T): Domain local pair natural orbital coupled cluster singles and doubles with perturbative triples
EOM-CCSD: Equation of motion coupled cluster singles and doubles
FGR: Fermi's golden rule
HEOM: Hierarchical equations of motion
MBAR: Multi-state Bennett acceptance ratio
MD: Molecular dynamics
*NPT*
: Constant particle number/pressure/temperature molecular dynamics
*NVE*
: Constant particle number/volume/energy molecular dynamics
*NVT*
: Constant particle number/volume/temperature molecular dynamics
WHAM: Weighted histogram analysis
SOCT: Spin–orbit coupled charge transfer
TDA: Tamm-Dancoff approximation
TDDFT: Time dependent density functional theory
TREPR: Time resolved electron paramagnetic resonance

## Data availability

OpenMM force field files and example scripts to run energy gap calculations, together with initial geometries, can be found at https://doi.org/10.5281/zenodo.10719345. Other data that is not available in the manuscript or ESI† is available from the corresponding authors upon a reasonable request.

## Author contributions

T. P. F.: conceptualization, data curation, formal analysis, investigation, software, writing – original draft, writing – review & editing (equal). D. T. L.: funding acquisition, supervision, writing – review & editing (equal).

## Conflicts of interest

The authors declare no conflict of interest.

## Supplementary Material

SC-015-D4SC01369G-s001
