# Supplementary material for: Unraveling the mechanisms of triplet state formation in a heavy-atom free photosensitizer
Source: Chem Sci. 2024 Apr 2;15(18):6726–37. doi: 10.1039/d4sc01369g (PMC11077524; doi:10.1039/d4sc01369g)
Supplement: SC-015-D4SC01369G-s001 [file SC-015-D4SC01369G-s001.pdf]

Electronic Supplementary Material (ESI) for Chemical Science.
This journal is © The Royal Society of Chemistry 2024

Supplementary Information: Unraveling the mechanisms of triplet state formation in a heavy-atom free photosensitizer

Thomas P. Fay[1, a)] and David T. Limmer[1, 2, 3, 4]
[1)] *Department of Chemistry, University of California Berkeley, CA 94720 USA*
[2)] *Kavli Energy Nanoscience Institute, Berkeley, CA 94720 USA*
[3)] *Chemical Science Division Lawrence Berkeley National Laboratory, Berkeley, CA 94720 USA*
[4)] *Material Science Division Lawrence Berkeley National Laboratory, Berkeley, CA 94720 USA*[b)]

CONTENTS

I. Methods and computational details 1
A. Electronic structure 1
B. Force field parameterization 2
C. Solvent models 3
D. Free energy calculations 3
E. Spin-boson mapping 4
F. Hierarchical equations of motion calculations 5

II. Intensity borrowing 5

III. Supplementary data 6
A. Solvent model properties 6
B. Gas phase energies 6
C. Couplings 6
D. Umbrella sampling 7
E. Reorganization energies 7

I. METHODS AND COMPUTATIONAL DETAILS

A. Electronic structure

Diabatic excited state force fields for solution-phase simulations were constructed based on gas phase electronic structure calculations. The geometry of each excited state of interest was first optimized using (TD)DFT at the ωB97X-D3/def2-SVP level of theory. Hessians were computed at these geometries for each of these excited states which were used in the parameterization of the force fields. Energy differences gas phase minimum energy geometries were computed using the wavefunction-based DLPNO-STEOM-CCSD method using the def2-TZVP(-f) basis set, except for the S_0–T_1 gap where the DLPNO-CCSD(T)/def2-TZVP(-f) was used.

The three singlet diabatic states were computed from the calculated using the multi-state generalization of the GMH method,wherein the dipole moment operator projected along the charge transfer axis, $\boldsymbol{e}_{\mathrm{CT}} \cdot \hat{\boldsymbol{\mu}}$, is first diagonalized, with its eigenvectors defining diabatic states. The 2×2 sub-block of resulting diabatic Hamiltonian corresponding the the S_0 and $^{\mathrm{S}}\mathrm{BD}^*$ states is then diagonalized to define the final (quasi-)diabatic Hamiltonian at each geometry. The resulting 3×3 quasi-diabatic Hamiltonian matrix has the form

$$\mathbf{H} = \begin{pmatrix} E_{\mathrm{S}_0} & 0 & V_{\mathrm{S}_0,{}^{\mathrm{S}}\mathrm{CT}} \\ 0 & E_{{}^{\mathrm{S}}\mathrm{BD}^*} & V_{{}^{\mathrm{S}}\mathrm{BD}^*,{}^{\mathrm{S}}\mathrm{CT}} \\ V_{\mathrm{S}_0,{}^{\mathrm{S}}\mathrm{CT}} & V_{{}^{\mathrm{S}}\mathrm{BD}^*,{}^{\mathrm{S}}\mathrm{CT}} & E_{{}^{\mathrm{S}}\mathrm{CT}} \end{pmatrix} \tag{S1}$$

Energies and dipole-moment operators were obtained from the DLPNO-STEOM-CCSD calculations. The charge transfer vector $\boldsymbol{e}_{\mathrm{CT}}$ is the unit vector in the direction $\boldsymbol{\mu}_{\mathrm{CT,ad}} - (\boldsymbol{\mu}_{\mathrm{S}_0,\mathrm{ad}} + \boldsymbol{\mu}_{{}^{\mathrm{S}}\mathrm{BD}^*,\mathrm{ad}})/2$, where $\boldsymbol{\mu}_{J,\mathrm{ad}}$ is the dipole moment of adiabatic state J.

a) Electronic mail: tom.patrick.fay@gmail.com
b) Electronic mail: dlimmer@berkeley.edu

For triplet-triplet couplings the mixed fragment-charge density/fragment excitation density method was used to compute diabatic states and spin-orbit couplings.[1] Löwdin charges and transition charge were computed using TDA-TDDFT ωB97X-D3/def2-TZVP at each of the excited state geometries with CPCM treatment of the ACN solvent. From these the fragment charge and excitation density operators were constructed for the adiabatic states corresponding to the states of interest, which were then diagonalized simultaneously using a Jacobi sweep algorithm.[2] Spin-orbit couplings were obtained using the TDDFT ωB97X-D3/def2-TZVPP/CPCM(ACN) using the SOMF method in Orca 5.0.3.[3,4]

B. Force field parameterization

A classical molecular mechanics description of the inter and intramolecular forces was employed for the different electronic states of BODIPY-Anth. For the intermolecular part, a non-polarizable fixed charge model was used for the electrostatic component together with a Lennard-Jones description of repulsion and dispersion effects. Charges for each state were parameterized by taking an average of CHELPG charges computed for each state at the SOS-ωB2G-GPLY/def2-TZVP(-f) level of theory, with and without CPCM treatment of the acetonitrile solvent as implemented in ORCA.[5,6] Lennard-Jones parameters were assumed to be the same for each electronic state and are taken from the OPLS-AA force field with boron parameters taken from Ref. 7.

The intramolecular forcefield is given as a sum of bonds, angles, torsion dihedrals and improper dihedrals, and bond-bond and bond-angle correlation terms, electrostatics and Lennard-Jones terms,

$$V = V_{\text{bond}} + V_{\text{angle}} + V_{\text{bond-bond}} + V_{\text{bond-angle}} + V_{\text{torsion}} + V_{\text{improper}} + V_{\text{el}} + V_{\text{LJ}} \tag{S2}$$

The long-range forces are ignored for 1-2 and 1-3 bonded atoms and scaled by 0.5 for 1-4 bonded atoms. Periodic dihedral and improper terms are used, as is standard for the parent OPLS-AA forcefield. Harmonic angle forces are used and bi-linear bond-bond and bond-angle terms were used for all 1-3 bonded triples of atoms,

$$V_{\text{bond-bond}} = \sum_{b,b' \neq b} k_{b,b'}(r_b - r_{b,0})(r_{b'} - r_{b',0}) \tag{S3}$$

$$V_{\text{bond-angle}} = \sum_{a,b} k_{a,b}(\theta_a - \theta_{a,0})(r_b - r_{b,0}) \tag{S4}$$

The $r_{b,0}$ and $\theta_{a,0}$ terms were set to the QM equilibrium geometry parameters. A Morse potential truncated at fourth order was used to describe bond stretches,

$$V_{\text{bond}} = \sum_b \frac{1}{2} k_b (r - r_{b,eq})^2 (1 - \alpha_b (r - r_{b,eq}) + \frac{7}{12}\alpha_b^2 (r - r_{b,eq})^2) \tag{S5}$$

with the Morse α parameter for each bond parameterized from Hessians at displaced geometries in the ground electronic state.

These potentials were parameterized by first fitting force-constants and equilibrium bond lengths/angles to the gas phase QM hessian calculated at the ωB97X-D3/def2-SVP equilibrium geometries, then refined bond lengths and bond angles to reproduce the gas phase equilibrium structures. The first step in parameterization after LJ parameters and charges were assigned was to fit intramolecular force field terms were by minimizing

$$\mathcal{L}_{\text{Hess}} = \sum_{A \geq B} ||\mathbf{H}_{AB,\text{MM}} - \mathbf{H}_{AB,\text{QM}}||^2 \tag{S6}$$

where $\mathbf{H}_{AB}$ denotes the partial hessian block. For this step the bonds are treated as harmonic. First the equilibrium bond lengths and angles are allowed to vary arbitrarily but in a second step they are fixed, with any lengths more than 0.0001 nm and angles more than 2.5° away from the QM equilibrium geometry values fixed at these boundaries. The equilibrium bond lengths and angles are then refined to minimize

$$\mathcal{L}_{\text{geom}} = \sum_{n=2,3,4} \sum_{A,B \in 1-n \text{ bonded pairs}} (r_{AB} - r_{AB,\text{QM}})^2. \tag{S7}$$

The improper and dihedral parameters were then refined again minimizing $\mathcal{L}_{\text{Hess}}$, and the geometry refinement was repeated after this. The forcefield obtained with the lowest value of $\mathcal{L}_{\text{geom}}$ was taken. The hessian fitting with $\mathcal{L}_{\text{Hess}}$ with multiple randomly displaced geometries was used to parameterize a re-scaling of the bond force constant k_b and the Morse α_b parameter for the ground-state. The same re-scalings and α_b parameters were then used for the excited states. Symmetry was used to constrain values of the force field parameters for equivalent atoms at each stage of the fitting.

For the polarizable solute model, Drude oscillators are placed on all non-hydrogen atoms. The atom polarizabilities and Thole parameters are fitted by fitting the molecular polarizability for the groundstate for each equilibroum state geometry from SOS-ωB2GP-PLYP/def2-TZVP(-f) calcualtions. We found that the poalrizability does not change considerably between charged states and singlet/triplet states of the individual components, so we consider this a reasonable approximation. Each atomic polarizability and Thole parameter was regularized vs values fitted to small symmetric molecules, namely C_6H_6 for aromatic atoms, C_2H_6 for the methyl groups, CF_4 for F and BF_4^- for B. In the fitting the CHELPG charges are simultaneously readjusted to optimally reproduce gas phase dipole and quadrupole moments, as well as the molecular polarizability tensor. In the fitting the charges are also regularised against the gas phase CHELPG values to avoid overfitting. This procedure cannot account for how charge may flow around the molecule when it is placed in a dielectric environment. To account for this, we also added the difference in density-derived Hirshfeld charges between vacuum and acetontrile CPCM calculations at the SOS-ωB2GP-PLYP/def2-TZVP(-f) level of theory to the final re-scaled charges obtained from the polarizability fitting.

C. Solvent models

Two acetonitrile force field models were parameterized to describe this system: a non-polarizable and a polarizable model. Both models were constructed by first performing hessian fitting to the RI-MP2/cc-pVQZ geometry and to obtain harmonic bond-stretch and angle-bend force constants. For the non-polarizable model Lennard-Jones parameters were taken from the OPLS-AA force field and charges were taken as a mixture of CPCM and gas phase CHELPG charges, $q_A = \lambda q_A^{\mathrm{CPCM}} + (1-\lambda) q_A^{\mathrm{gas}}$, with the mixing parameter λ optimized to reproduce the experimental density at 298 K.

For the polarizable model a single Drude oscillator was added to the nitrile group carbon atom, with an anisotropic polarizability obtained from a RI-MP2/cc-pVQZ calculation. A modified version of the three-step parameterization procedure was then followed to parameterize charges, Lennard-Jones ϵ parameters and σ parameters.[8] First the charges were re-scaled according to $q_A = \lambda q_A^{\mathrm{CPCM}} + (1-\lambda) q_A^{\mathrm{gas}}$ in order to reproduce the experimental dielectric constant at 298 K. Second, the ϵ parameters in the Lennard-Jones potential were re-scaled to reproduce the vaporization enthalpy at 298 K, and third σ parameters were re-scaled to reproduce the experimental density at 298 K. The resulting force-fields both accurately reproduce these properties of ACN at 298 K, as well as the dielectric relaxation time.

All fitted free energy curves shown in the main text are fitted by fitting a polynomial approximation to the free energy curves for A, $\tilde{A}_{A,n}(\epsilon) = \sum_{k=0}^{n} a_n \epsilon^n$, and B, $\tilde{A}_{B,n}(\epsilon) = \tilde{A}_{A,n}(\epsilon) + \epsilon - \Delta A_{A\to B}$, to to cumulative distribution function,

$$\mathrm{CDF}_J(\epsilon) = \int_{-\infty}^{\epsilon} \mathrm{d}\epsilon' \, p_J(\epsilon') \tag{S8}$$

for $J = A$ and B. The constraint the the integral of $\tilde{p}_J(\epsilon) = 1$ is also added in the fitting. For the fits to the umbrella-sampled free energy curves we only fit to the A curve.

D. Free energy calculations

The free energy change for the transformation from electronic state A to B was calculated for the non-polarizable model using thermodynamic integration runs, where the potential is given by $V_\lambda(\boldsymbol{q}) = (1-\lambda)V_A(\boldsymbol{q}) + \lambda V_B(\boldsymbol{q})$. Five MD simulations were performed for each transformation with λ values equally spaced between 0 and 1. The Fast-Forward Langevin integrator was used to sample configurations with a time-step of 1 fs.[9] MBAR was used to compute the Helmholtz free energy change $\Delta A_{A\to B}$ from these thermodynamic integration runs. WHAM was used with these runs to compute free energy curves and free energy barriers for each electron transfer process. For the $^{\mathrm{S}}\mathrm{CT} \longrightarrow \mathrm{S}_0$ and $^{\mathrm{T}}\mathrm{BD}^* \longrightarrow \mathrm{S}_0$ processes the crossing region for the two diabatic states was not sampled in the thermodynamic integration runs so umbrella sampling was performed with the the diabatic energy gap $\Delta V = V_B(\boldsymbol{q}) - V_A(\boldsymbol{q})$ used as a biasing coordinate, and again WHAM was used to obtain free energy curves and the free energy barrier for this electron transfer. All fast-forward Langevin dynamics simulations were performed using an in-house code using the OpenMM 7 C++ library for force and energy evaluations.[10]

The box containing BD−An was set-up with 512 ACN molecules, which was found to provide adequately almost completely decayed dipole-dipole correlations, using Packmol.[11] Three independent NPT trajectories were run with the S_0 BD−An force field and the non-polarizable ACN model to find an equilibrated box side length of $d = 3.570\,\mathrm{nm}$. In the thermodynamic integration five equally spaced windows were sampled, with each window sampled for 1 ns after 0.1 ns of equilibration with a FF-Langevin friction constant of $\gamma = 4\,\mathrm{ps}^{-1}$ and a time-step of 1 fs. For umbrella sampling the same simulation parameters and trajectory lengths were used, with an umbrella sampling force constant of $k_U = 0.01\,\mathrm{kJ}^{-1}\,\mathrm{mol}$ with windows centered at integer multiples of $50\,\mathrm{kJ\,mol}^{-1}$.

For the polarizable model free energy changes were obtained from MBAR with configurations sampled on each electronic state. Free energy barriers were obtained by fitting free energy curves obtained from MBAR to a polynomial. For the charge

transfer steps a quadratic polynomial was used but for other processes anharmonicity plays a larger role and a higher order order polynomial was used. Error bars were obtained by bootstrapping the six independent runs. All simulations were performed using the python API of OpenMM 7 and were analyzed with in-house scripts. For the NVT runs, trajectories were equilibrated for 5 ps and sampled for 2 ns using the extended Lagrangian approach, with a time-step of 1 fs, a friction coefficient of $\gamma = 2\,\mathrm{ps}^{-1}$ for the centers of mass and $20\,\mathrm{ps}^{-1}$ for the Drude oscillators, with the Drude particle temperature set to 1 K.

E. Spin-boson mapping

For each non-adiabatic process a spin-boson mapping was constructed where an effective potential for each state is constructed as

$$V_J^{\mathrm{SB}}(\boldsymbol{x}) = \sum_\alpha \left(\frac{1}{2} m_\alpha \omega_\alpha^2 x_\alpha^2 + \delta_{B,J} c_\alpha x_\alpha\right) + \delta_{B,J}(\Delta A_{A\to B} + \lambda) \tag{S9}$$

The reorganization energy λ was fitted to reproduce the free energy barrier to reach the crossing point for $A \to B$, as obtained from the free energy calculations. The rate constants and spectra for the spin-boson mapping depend only on the spectral density

$$\mathcal{J}(\omega) = \frac{\pi}{2}\sum_\alpha \frac{c_\alpha^2}{m_\alpha \omega_\alpha}\delta(\omega - \omega_\alpha). \tag{S10}$$

This can be parameterized directly from the Fourier transform of the energy gap correlation function obtained from classical MD. In what follows, we parameterize this as

$$\mathcal{J}(\omega) = \pi\lambda\omega\rho(\omega) \tag{S11}$$

where the spectral distribution $\rho(\omega)$ is calculated from MD as

$$\rho_J(\omega) = \frac{\frac{2}{\pi}\int_0^\infty \cos(\omega t)\,\langle \delta\Delta V(t)\delta\Delta U(0)\rangle_J\,\mathrm{d}t}{\langle \delta\Delta V^2\rangle_J}. \tag{S12}$$

where $\Delta V = V_B - V_A$, $\delta\Delta V = \Delta V - \langle\Delta V\rangle_J$ and $\langle\cdots\rangle_J$ denotes the classical phase space average over the equilibrium distribution for state J with dynamics calculated on the same surface. By fitting the reorganization energy based off of the free energy barrier we ensure the spin-boson mapping we construct becomes exact in the high-temperature limit. There are two possible $\rho(\omega)$ distributions for each transformation, obtained from dynamics on either A or B, and in general these distributions will differ. The similarity of the distributions, and in particular the quantities of interest computed from the two different distributions, gives an indication of how accurate the spin-boson mapping can be expected to be. For all computed rate constants, we use an average of rate constants computed with $\rho_A(\omega)$ and $\rho_B(\omega)$, although we have found that first averaging the spectral distributions and computing rates from this averaged distribution yields almost identical final rate constants.

The correlation function appearing in the full FGR rate constant can be evaluated as

$$\begin{aligned}&\frac{\mathrm{Tr}\left[e^{-\beta\hat{H}_A}e^{-i\hat{H}_A t/\hbar}e^{+i\hat{H}_B t/\hbar}\right]}{\mathrm{Tr}\left[e^{-\beta\hat{H}_A}\right]}\\ &= \exp\left(-i\frac{\Delta E_{\mathrm{AB}}t}{\hbar} - \frac{1}{\pi\hbar}\int_0^\infty \frac{\mathcal{J}(\omega)}{\omega^2}\left[\coth\left(\frac{\hbar\omega}{2k_BT}\right)(1-\cos(\omega t)) - i\sin(\omega t)\right]\mathrm{d}\omega\right),\end{aligned} \tag{S13}$$

where $\Delta E_{AB} = -\Delta A_{A\to B}$. This is evaluated by discretizing the spectral density using the method in Ref. 12. The spectra can also be directly obtained from the Fourier transform of this function. The correlation function is multiplied by an exponential decay $e^{-t/\tau}$ when computing the spectra to account for instrument broadening. A time-constant $\tau = 350\,\mathrm{fs}$ is used, as has been used in other studies.

Spectral densities for the non-polarizable model were obtained from 20 NVE trajectories each 30 ps long with a time step of 0.5 fs. The cross correlated portion of the spectral distribution was found to have a negligible effect of the calculated rate constants (as long as the total reorganization energy was held fixed at the fitted value). Furthermore, given that the Debye relaxation time for the non-polarizable and polarizable models are almost identical (see table S1) and that this model very accurately describes the outer sphere contribution, we constructed the polarizable model spectral distribution as

$$\rho^{\mathrm{pol}}(\omega) = (1 - f_{\mathrm{inner}}^{\mathrm{pol}})\rho_{\mathrm{outer}}^{\mathrm{non-pol}}(\omega) + f_{\mathrm{inner}}^{\mathrm{pol}}\rho_{\mathrm{inner}}^{\mathrm{non-pol}}(\omega). \tag{S14}$$

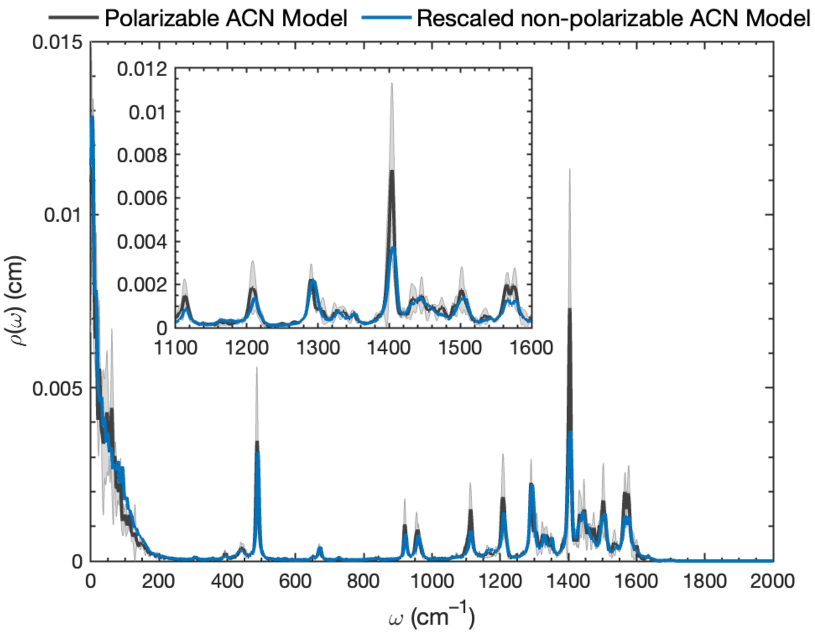

Fig. S1. Full polarizable ACN spectral distribution and the approximate form given by Eq. (S14) for the ^{S}CT → S_0 process, calculated with dynamics on the ^{S}CT surface.

This avoids the need to run expensive self-consistent field Drude oscillator calculations to obtain the spectral density for the non-polarizable model. The proportion of inner sphere reorganization energy was set as

$$f_{\rm inner} = \frac{1}{2}\frac{\langle \Delta V_{\rm mol}\rangle_B - \langle \Delta V_{\rm mol}\rangle_A}{\lambda_{\rm LR}}. \tag{S15}$$

which was found to agree well with values obtained from the co-variance based estimates. In Fig. S1 we show the approximated spectral distribution based off of the re-scaled non-polarizable ACN calculations to the full spectral distribution calculated from three NVE trajectories with the full polarizable ACN potential with SCF Drude oscillator integration. We see excellent agreement (within the uncertainty of the polarizable model simulation) between the approximate and full spectral distribution.

The coupling H_{AB} for the rate constant calculation was taken as the root-mean-square of the couplings between the two gas-phase equilibrium geometries of A and B, i.e. $H_{AB} = \sqrt{(|H_{AB,A}|^2 + |H_{AB,B}|^2)/2}$. This ensures a symmetric definition of the rate constants, but does not account for non-Condon effects.

F. Hierarchical equations of motion calculations

The hierarchical equations of motion (HEOM) method, as implemented in the `heom-lab` Matlab code[13,14] was used to obtain exact rate constants for the model spectral density for the ^{S}CT→S_0 transition. The system was initialised in the state $\hat{\rho}(0) = |A\rangle\langle A|\,\hat{\rho}_{\rm b}$ and the long time dynamics of the B state population were fitted to a function

$$p_B(t) = (p_\infty - p_0)(e^{-kt} - 1) + p_0 \tag{S16}$$

in order to obtain the rate constant. The calculations used the HEOM truncation scheme from Ref. 15 with $L = 80$, $L\Gamma = 6$ and a Matsurbara expansion was used for the bath correlation functions with the $M = 1$ Matsubara mode included explicitly in the calculations. The remaining Matsubara terms were treated with the low temperature correction scheme in Ref. 14. Dynamics were run out to $t = 10\hbar\beta$.

II. INTENSITY BORROWING

The effective transition dipole moments used in the spectral calculation are taken directly from the adiabatic DLPNO-STEOM-CCSD calculations. From the diabatization we can analyze the origin of the relatively large transition dipole moment for the ^{S}CT

state. The adiabatic states are given by first order perturbation theory in the diabatic couplings H_{AB} as

$$|\Psi_A\rangle = |A\rangle + \sum_{J\neq A} |J\rangle \frac{H_{A,J}}{E_A - E_J} \tag{S17}$$

From this we find the mixing coefficient for the ^{S}CT and S_0 states is 0.12 compared to 0.053 for the ^{S}CT and ^{S}BD* states. The transition dipole moment is given to lowest order in perturbation theory by

$$\langle\Psi_A|\hat{\mu}|\Psi_B\rangle = \langle A|\hat{\mu}|B\rangle + \sum_{J\neq A} \langle J|\hat{\mu}|B\rangle \frac{H^*_{A,J}}{E_A - E_J} + \sum_{J\neq A} \langle A|\hat{\mu}|J\rangle \frac{H_{B,J}}{E_B - E_J} \tag{S18}$$

and for the ^{S}CT $\longrightarrow$ S_0 transition this is given approximately by

$$\begin{aligned} \left\langle\Psi_{^S\mathrm{CT}}|\hat{\mu}|\Psi_{\mathrm{S}_0}\right\rangle = {} & \left\langle{}^S\mathrm{CT}|\hat{\mu}|\mathrm{S}_0\right\rangle + \left(\langle \mathrm{S}_0|\hat{\mu}|\mathrm{S}_0\rangle - \left\langle{}^S\mathrm{CT}|\hat{\mu}|{}^S\mathrm{CT}\right\rangle\right) \frac{H_{^S\mathrm{CT},\mathrm{S}_0}}{E_{^S\mathrm{CT}} - E_{\mathrm{S}_0}} \\ & + \left\langle{}^S\mathrm{BD}^*|\hat{\mu}|\mathrm{S}_0\right\rangle \frac{H_{^S\mathrm{CT},{}^S\mathrm{BD}^*}}{E_{^S\mathrm{CT}} - E_{^S\mathrm{BD}^*}}. \end{aligned} \tag{S19}$$

The second term in this expression, arising from mixing of the ^{S}CT and S_0 states dominates. Thus it is primarily ^{S}CT-S_0 mixing that enables intensity borrowing in the ^{S}CT emission.

III. SUPPLEMENTARY DATA

A. Solvent model properties

In table S1 we show a summary of solvent properties calculated from molecular dynamics for the solvent models. All simulations used a box of 512 ACN molecules. All simulations were performed at 298 K. These properties were considered as good target properties for accurate simulations of electron transfer in ACN at 298 K.

Property	Non-polarizable	Polarizable (eV)	Experiment
Density (g cm^{-1})	0.7758 ± 0.0001	0.7779 ± 0.0002	0.7778[16]
ΔH_{vap} (kJ mol^{-1})	39.2 ± 0.3	34.2 ± 0.4	33.225[17]
Static dielectric constant, ϵ_0	29.8 ± 2.7	37.5 ± 2.8	35.55[18]
Optical dielectric constant, $\epsilon_\infty = n^2$	1	1.75	1.81[19]
Dielectric relaxation time, τ_{rel} (ps)	4.1	3.0	3.3[18]
Debye time, $\tau_D = (\epsilon_\infty/\epsilon_0)\tau_{\mathrm{rel}}$ (ps)	0.141	0.140	0.168

Table S1. Physical properties of ACN calculated for the polarizable and non-polarizable models and experimental values for comparison.

B. Gas phase energies

A comparison of gas phase minimum energy geometry energies, calculated with different basis sets, relative to the S_0 state energy is shown in table S2.

C. Couplings

We have computed both the spin-conserving diabatic couplings as well as spin-orbit couplings between singlet and triplet states. Table S3 shows the computed values of these H_{AB} at the minimum energy geometries of state A and B. The spin-orbit couplings are of course significantly smaller than the spin-conserving diabatic couplings. We note that couplings between the ^{S}CT and S_0 state are significantly larger than the ^{S}CT-^{S}BD* couplings, and the SOC couplings are all small, with the coupling for the El-Sayed's rule forbidden ^{S}CT$\longrightarrow$^{T}CTtransition not being significantly smaller than the other SOC mediated charge transfer processes, despite there not being a change in orbital occupancy between these states. This can likely be attributed to the fact that although the SOC-mediated CT processes involve a change in orbital occupancy, the orbitals involved are localised on different fragments of the molecule, so the poor orbital overlap reduces the SOC between these states. Overall we see that all three ^{S}CT$\longrightarrow$$T_n$ pathways, which are all feasible according to the free energy changes, are weakly allowed by the SOC interaction.

State	def2-SVP (eV)	def2-TZVP(-f) (eV)	Shifted def2-TZVP(-f)
S_0	0	0	0
$^{S}BD^*$	2.232	2.389	2.478
^{S}CT	2.780	2.927	3.017
$^{T}CT^{a}$	2.872	3.025	3.114
$^{T}An^{*b}$	–	2.017	2.017
$^{T}BD^{*b}$	1.593	1.558	1.558

Table S2. Gas phase energies of the different excited states of BD – An at their respective minimum energy geometries (from gas phase TDA-TDDFT ωB97X-D3/def2-SVP geometry optimizations) computed at the DLPNO-STEOM-CCSD level of theory with different basis sets. [a]Geometry obtained from calculation with CPCM treatment of ACN solvent. [b]Energies computed from $S = 1$ ground state DLPNO-CCSD(T) calculations.

D. Umbrella sampling

For the $^{S}CT \rightarrow S_0$ and $^{T}BD^* \rightarrow S_0$ processes the transitions lie very deep in the Marcus inverted regime, which means that enhanced sampling is required to obtain accurate free energy profiles. We have performed Umbrella sampling directly on the energy gap coordinate ΔV for these two transitions, in order to sample rare fluctuations of the solvent and molecule in which $\Delta V = 0$ and subsequently compute the free energy barrier. Efficient molecular dynamics sampling on the biased potentials with the polarizable ACN model is not possible, because the extended Lagrangian approach cannot be applied, so we only use the non-polarizable ACN model in these simulations. In Fig. S2 we show these free energy curves, together with a quadratic fit to the A state curve data (obtained by fitting the corresponding cumulative distribution function, see SI for details), excluding the portion of the curve where the energy gap, ϵ, is less than 1 eV. We see that in the $^{S}CT \rightarrow S_0$ (Fig. S2A) the quadratic fit provides an excellent fit, which extrapolates accurately to the crossing point. The effective fitted Marcus reorganization energy, λ_{fit}, obtained by equating the Marcus-theory (Gaussian) probability density at the crossing point and the Umbrella sampling probability density, agree very well, with $\lambda_{\text{fit}} = 0.590$ eV compared to the quadratic estimate $\lambda_{\text{quadratic}} = 0.585$ eV. This suggests that obtaining λ_{fit} for the polarizable model by fitting the free energy curves (which do not sample the $\epsilon = 0$ region) to a quadratic and extrapolating should introduce an error of only $\simeq 0.005$ eV. In stark contrast, for the $^{T}BD^*$and S_0 states there is a large deviation between a quadratic fit and the computed free energy curves for the $^{T}BD^* \rightarrow S_0$ process (Fig. S2B). This can be attributed to the fact that $\sim 98\%$ of the reorganization energy for the $^{T}BD^* \rightarrow S_0$ arises from intra-molecular inner sphere contributions (see below for how this is calculated), for which asymmetry in the vibrational frequencies and anharmonicity in the molecular potential energy surfaces plays a significant role. However for the $^{S}CT \rightarrow S_0$ process the inner sphere contribution is only about 40% of the total reorganization energy, so the outer sphere contribution arising from fluctuations in the solvent polarization dominates, which is very well described by a Gaussian field theory.

E. Reorganization energies

It is interesting to quantify how different the reorganization energies are between the different state transitions, but as observed above, not all of the free energy curves are perfectly quadratic. However, as stated above, we can fit $p_A(\epsilon = 0)$ to the Gaussian functional form in Marcus theory $p_A^{G}(\epsilon) = (4\pi\lambda k_B T)^{-1/2} e^{-(\Delta A_{A\rightarrow B} - \epsilon + \lambda)^2 / 4\lambda k_B T}$, to obtain a effective reorganization energy λ_{fit}. Predictably it is smallest for the $^{S}CT \rightarrow {}^{T}CT$ transition, which involves no change in orbital occupancy or significant charge

A	B	$\lvert H_{AB}\rvert$ at A (cm^{-1})	$\lvert H_{AB}\rvert$ at B (cm^{-1})
$^{S}BD^*$	^{S}CT	64^{a}	125^{a}
S_0	^{S}CT	865^{a}	2550^{a}
^{S}CT	^{T}CT	0.28^{a}	0.11^{a}
^{S}CT	$^{T}An^*$	0.62^{b}	0.64^{b}
^{S}CT	$^{T}BD^*$	0.79^{b}	0.80^{b}
^{T}CT	$^{T}An^*$	505^{c}	351^{c}
^{T}CT	$^{T}BD^*$	12^{c}	53^{c}
$^{T}An^*$	$^{T}BD^*$	0.23^{c}	3.6^{c}
S_0	$^{T}BD^*$	0.17^{b}	0.21^{b}

Table S3. Couplings between different states in BD – An. [a] GMH couplings from DLPNO-STEOM-CCSD/def2-SVP. [b] SOMF couplings from TDDFT ωB97X-D3/def2-TZVPP/CPCM(ACN) spin orbiot couplings ($H_{AB}^2 = \sum_{\alpha=x,y,z} |H_{AB,\alpha}|^2$ where α denotes component of the SOC operator).[3] [c] FED/FCD couplings[1] from TDA-TDDFT ωB97X-D3/def2-TZVP/CPCM(ACN).

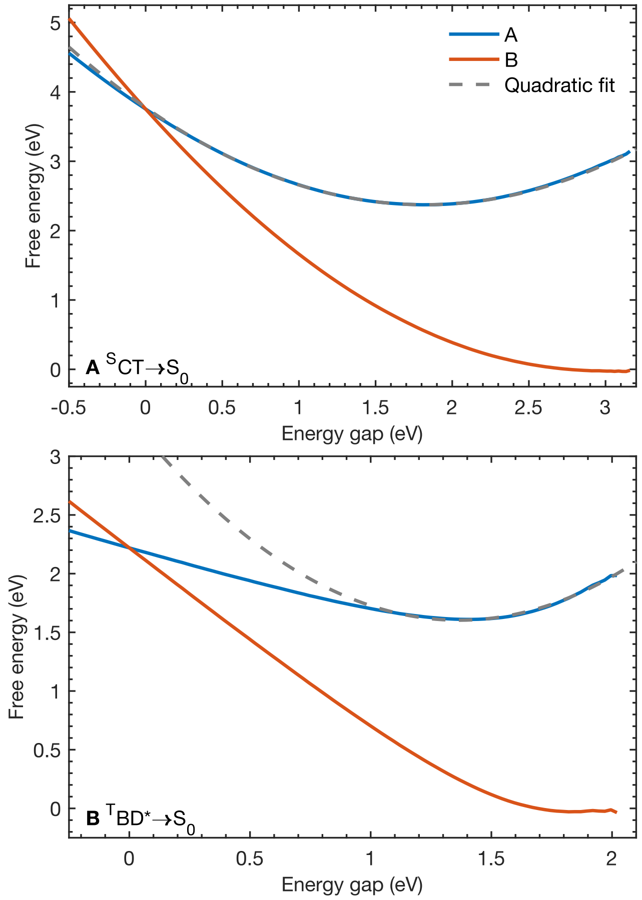

Fig. S2. Free energy curves for the ^{S}CT→S_0 (A) and ^{T}BD*→S_0 (B) processes obtained from umbrella sampling on the ^{S}CT and ^{T}BD* surfaces. The dashed line shows a quadratic fit to the curves with data c at energy gaps > 1 eV, i.e. close to the A minimum.

redistribution, at 0.11 eV.

In order to further investigate the role of non-Gaussian fluctuations in transitions between different excited states, we have computed several other estimates of the the Marcus theory reorganization energy for each of the processes. In addition to the fitted value, we can also estimate the reorganization energy from linear-response theory as[12,20]

$$\lambda_{\mathrm{LR}} = \frac{1}{2}\left(\langle \Delta V\rangle_A - \langle \Delta V\rangle_B\right) \tag{S20}$$

or from the energy gap fluctuations on a given surface $J = A$ or B,[12,20]

$$\lambda_{\mathrm{var},J} = \frac{\left\langle (\Delta V - \langle \Delta V\rangle_J)^2\right\rangle_J}{2k_{\mathrm{B}}T} = \frac{\left\langle \delta\Delta V^2\right\rangle_J}{2k_{\mathrm{B}}T}. \tag{S21}$$

If the linear response approximation in Marcus theory is valid, then all of these estimates will agree, and therefore deviations between these estimates are indicative of non-Gaussian effects. We see that for the ^{S}BD*→^{S}CT and ^{S}CT→S_0 the four estimates are in very close agreement, but for the other processes there are more significant deviations between the estimates, although these deviations are still relatively small. The largest deviations are for the processes dominated by the inner-sphere contribution to the reorganization energy, namely the ^{S}CT→^{T}CT, ^{T}An*→^{T}BD* and ^{T}BD*→S_0 processes, which is unsurprising given the greater importance of asymmetric vibrational frequencies and anharmonicity in the inner sphere contribution. We see however that the linear-response estimate for the reorganization energy generally agrees very well with the fitted value, which suggests that asymmetry in the energy gap fluctuations between surfaces A and B contribute primarily to the free energy differences between these states, and they have only a small effect on the barrier height.

The variance based estimators for the reorganization energy also enable us to decompose the reorganization energy in inner sphere, outer sphere, and inner-outer-sphere cross-correlation contributions. We decompose the energy gap ΔV into an intramolecular contribution and a remaining environment contribution, $\Delta V = \Delta V_{\mathrm{mol}} + \Delta V_{\mathrm{env}}$. We take the molecular contribution

	A	B	λ_{fit}/eV	λ_{LR}/eV	$\lambda_{\text{var,A}}$/eV	$\lambda_{\text{var,B}}$/eV	$f_{\text{var}}^{\text{inner}}$	$\lambda_{\text{var}}^{\text{inner}}$/eV	$\lambda_{\text{var}}^{\text{outer}}$/eV	$\lambda_{\text{var}}^{\text{cross}}$/eV
Polarizable ACN	^{S}BD*	^{S}CT	0.550	0.550	0.557	0.525	0.436	0.235	0.235	-0.008
	^{S}CT	S_0	0.483	0.482	0.464	0.471	0.453	0.212	0.212	0.001
	^{S}CT	^{T}CT	0.119	0.125	0.132	0.169	0.891	0.134	0.134	0.001
	^{S}CT	^{T}An*	0.477	0.478	0.433	0.487	0.494	0.227	0.227	-0.002
	^{S}CT	^{T}BD*	0.565	0.567	0.598	0.613	0.997	0.604	0.604	-0.004
	^{T}An*	^{T}BD*	0.584	0.584	0.547	0.618	0.507	0.294	0.294	-0.011
	^{T}BD*	S_0	–	0.225	0.333	0.206	0.967	0.261	0.261	-0.002
Non-pol ACN	^{T}BD*	S_0	0.512	0.236	0.346	0.239	0.98	0.283	0.008	-0.002
	^{S}CT*	S_0	0.590	0.581	0.603	0.590	0.40	0.233	0.345	0.002

Table S4. Uncertainties in the reorganization energies (2 standard errors in mean obtained from bootstrapping data NVT runs initialized from independent initial configurations) are all < 0.005 eV.

to be the difference in energies at a given configuration in the absence of the solvent, and the environment term as the remainder $\Delta V_{\text{env}} = \Delta V_{\text{mol}} - \Delta V$, which is effectively the difference in solvation energies between the two states A and B. This allows us to decompose the reorganization energy as $\lambda_{\text{var},J} = \lambda_{\text{var},J}^{\text{inner}} + \lambda_{\text{var},J}^{\text{outer}} + 2\lambda_{\text{var},J}^{\text{cross}}$, with the contributions given by[20]

$$\lambda_{\text{var},J}^{\text{inner}} = \frac{\langle \delta\Delta V_{\text{mol}}^2 \rangle_J}{2k_{\text{B}}T}, \quad \lambda_{\text{var},J}^{\text{outer}} = \frac{\langle \delta\Delta V_{\text{env}}^2 \rangle_J}{2k_{\text{B}}T}$$
$$\lambda_{\text{var},J}^{\text{cross}} = \frac{\langle \delta\Delta V_{\text{mol}} \delta\Delta V_{\text{env}} \rangle_J}{2k_{\text{B}}T}. \tag{S22}$$

We see that variations in the total reorganization energy arise from variations in both the inner and outer sphere contributions. For the charge transfer processes the inner sphere contribution is typically slightly smaller than the outer sphere contribution, but for transitions which are not accompanied by charge transfer the inner sphere contribution makes up over 90% of the total reorganization energy. We also see that for all transitions the cross correlation contributions are very small compared to the total reorganization energy.

The spectral distributions for the various photophysical processes are given in Fig. S3.

[1] J. Tölle, L. Cupellini, B. Mennucci, and J. Neugebauer, "Electronic couplings for photo-induced processes from subsystem time-dependent density-functional theory: The role of the diabatization," The Journal of Chemical Physics **153**, 184113 (2020).
[2] Y.-C. Wang, S. Feng, W. Liang, and Y. Zhao, "Electronic Couplings for Photoinduced Charge Transfer and Excitation Energy Transfer Based on Fragment Particle–Hole Densities," The Journal of Physical Chemistry Letters **12**, 1032–1039 (2021).
[3] F. Neese, "Efficient and accurate approximations to the molecular spin-orbit coupling operator and their use in molecular g-tensor calculations," The Journal of Chemical Physics **122**, 034107 (2005).
[4] B. De Souza, G. Farias, F. Neese, and R. Izsák, "Predicting Phosphorescence Rates of Light Organic Molecules Using Time-Dependent Density Functional Theory and the Path Integral Approach to Dynamics," Journal of Chemical Theory and Computation **15**, 1896–1904 (2019).
[5] H. S. Muddana, N. V. Sapra, A. T. Fenley, and M. K. Gilson, "The SAMPL4 hydration challenge: evaluation of partial charge sets with explicit-water molecular dynamics simulations," Journal of Computer-Aided Molecular Design **28**, 277–287 (2014).
[6] M. Jorge, M. C. Barrera, A. W. Milne, C. Ringrose, and D. J. Cole, "What is the Optimal Dipole Moment for Nonpolarizable Models of Liquids?" Journal of Chemical Theory and Computation **19**, 1790–1804 (2023).
[7] B. Doherty, X. Zhong, S. Gathiaka, B. Li, and O. Acevedo, "Revisiting OPLS Force Field Parameters for Ionic Liquid Simulations," Journal of Chemical Theory and Computation **13**, 6131–6145 (2017).
[8] E. Núñez-Rojas, V. García-Melgarejo, A. Pérez De La Luz, and J. Alejandre, "Systematic parameterization procedure to develop force fields for molecular fluids using explicit water," Fluid Phase Equilibria **490**, 1–12 (2019).
[9] M. Hijazi, D. M. Wilkins, and M. Ceriotti, "Fast-forward Langevin dynamics with momentum flips," The Journal of Chemical Physics **148**, 184109 (2018).
[10] P. Eastman, J. Swails, J. D. Chodera, R. T. McGibbon, Y. Zhao, K. A. Beauchamp, L.-P. Wang, A. C. Simmonett, M. P. Harrigan, C. D. Stern, R. P. Wiewiora, B. R. Brooks, and V. S. Pande, "OpenMM 7: Rapid development of high performance algorithms for molecular dynamics," PLOS Computational Biology **13**, e1005659 (2017).
[11] L. Martínez, R. Andrade, E. G. Birgin, and J. M. Martínez, "P ACKMOL : A package for building initial configurations for molecular dynamics simulations," Journal of Computational Chemistry **30**, 2157–2164 (2009).
[12] J. E. Lawrence and D. E. Manolopoulos, "Confirming the role of nuclear tunneling in aqueous ferrous–ferric electron transfer," The Journal of Chemical Physics **153**, 154114 (2020).
[13] T. P. Fay, "`heom-lab`: A Matlab code for performing HEOM calculations," `https://github.com/tomfay/heom-lab` (2022).
[14] T. P. Fay, "A simple improved low temperature correction for the hierarchical equations of motion," The Journal of Chemical Physics **157**, 054108 (2022).
[15] L. P. Lindoy, A. Mandal, and D. R. Reichman, "Quantum dynamical effects of vibrational strong coupling in chemical reactivity," Nature Communications **14**, 2733 (2023).
[16] D. F. Grant-Taylor and D. D. Macdonald, "Thermal pressure and energy–volume coefficients for the acetonitrile + water system," Canadian Journal of Chemistry **54**, 2813–2819 (1976).
[17] W. E. Putnam, D. M. McEachern, and J. E. Kilpatrick, "Entropy and Related Thermodynamic Properties of Acetonitrile (Methyl Cyanide)," The Journal of Chemical Physics **42**, 749–755 (1965).
[18] M. L. T. Asaki, A. Redondo, T. A. Zawodzinski, and A. J. Taylor, "Dielectric relaxation and underlying dynamics of acetonitrile and 1-ethyl-3-methylimidazolium triflate mixtures using thz transmission spectroscopy," The Journal of Chemical Physics **116**, 10377–10385 (2002).

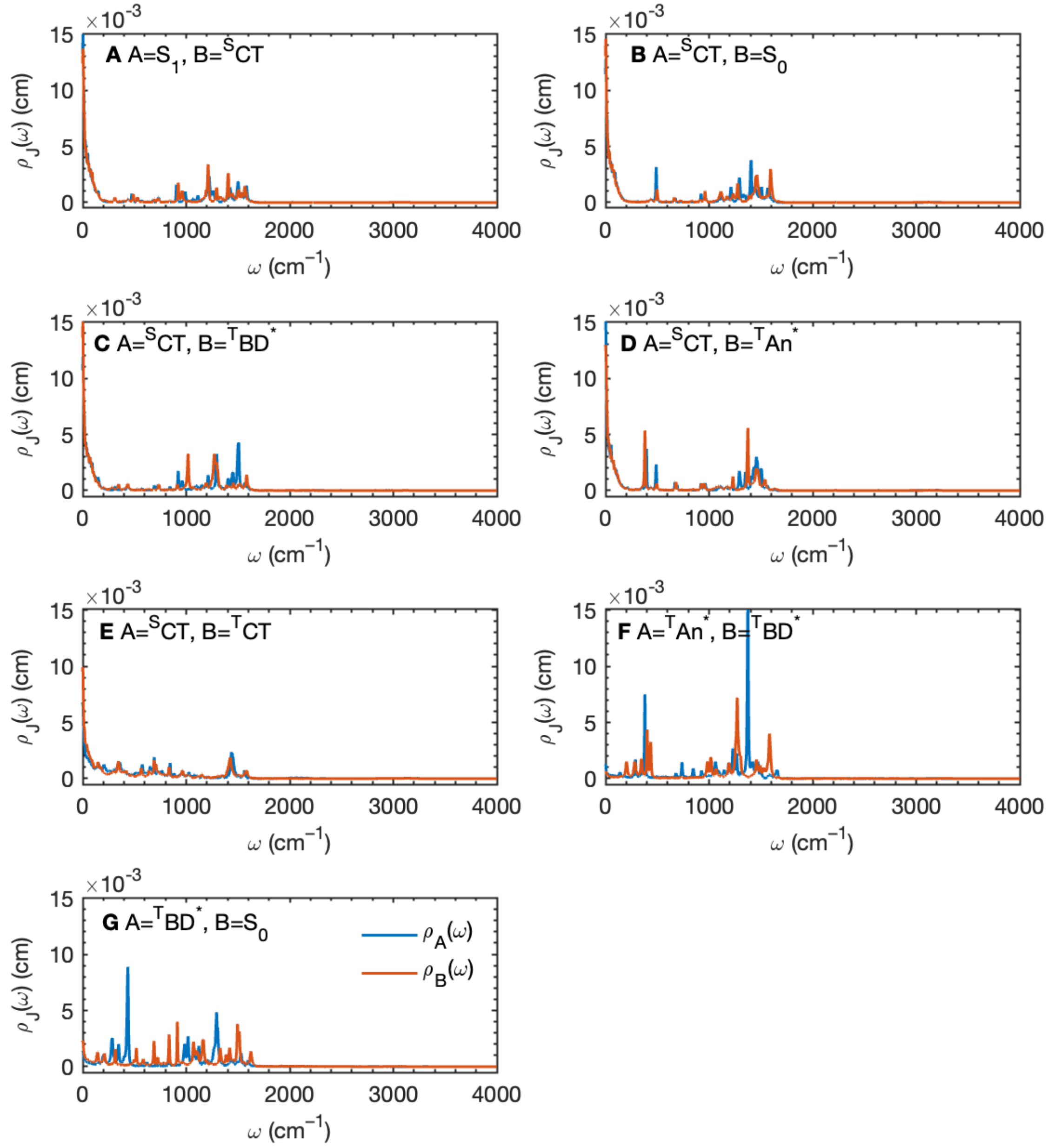

Fig. S3. Spectral densities for the processes considered in this work.

[19] K. Moutzouris, M. Papamichael, S. C. Betsis, I. Stavrakas, G. Hloupis, and D. Triantis, "Refractive, dispersive and thermo-optic properties of twelve organic solvents in the visible and near-infrared," Applied Physics B **116**, 617–622 (2014).

[20] J. Blumberger and G. Lamoureux, "Reorganization free energies and quantum corrections for a model electron self-exchange reaction: comparison of polarizable and non-polarizable solvent models," Molecular Physics **106**, 1597–1611 (2008).
